# Sensitive, High-Throughput HLA-I and HLA-II Immunopeptidomics Using Parallel Accumulation-Serial Fragmentation Mass Spectrometry

**DOI:** 10.1016/j.mcpro.2023.100563

**Published:** 2023-05-03

**Authors:** Kshiti Meera Phulphagar, Claudia Ctortecka, Alvaro Sebastian Vaca Jacome, Susan Klaeger, Eva K. Verzani, Gabrielle M. Hernandez, Namrata D. Udeshi, Karl R. Clauser, Jennifer G. Abelin, Steven A. Carr

**Keywords:** HLA-I and II immunopeptidomics, high-throughput acquisition, trapped ion mobility, sensitive single-shot MS analysis

## Abstract

Comprehensive and in-depth identification of the human leukocyte antigen class I (HLA-I) and class II (HLA-II) tumor immunopeptidome can inform the development of cancer immunotherapies. Mass spectrometry (MS) is a powerful technology for direct identification of HLA peptides from patient-derived tumor samples or cell lines. However, achieving sufficient coverage to detect rare and clinically relevant antigens requires highly sensitive MS-based acquisition methods and large amounts of sample. While immunopeptidome depth can be increased by off-line fractionation prior to MS, its use is impractical when analyzing limited amounts of primary tissue biopsies. To address this challenge, we developed and applied a high-throughput, sensitive, and single-shot MS-based immunopeptidomics workflow that leverages trapped ion mobility time-of-flight MS on the Bruker timsTOF single-cell proteomics system (SCP). We demonstrate greater than twofold improved coverage of HLA immunopeptidomes relative to prior methods with up to 15,000 distinct HLA-I and HLA-II peptides from 4e7 cells. Our optimized single-shot MS acquisition method on the timsTOF SCP maintains high coverage, eliminates the need for off-line fractionation, and reduces input requirements to as few as 1e6 A375 cells for >800 distinct HLA-I peptides. This depth is sufficient to identify HLA-I peptides derived from cancer-testis antigen and noncanonical proteins. We also apply our optimized single-shot SCP acquisition methods to tumor-derived samples, enabling sensitive, high-throughput, and reproducible immunopeptidome profiling with detection of clinically relevant peptides from less than 4e7 cells or 15 mg wet weight tissue.

## Highlights

• HLA-I and HLA-II peptides increased >2-fold using trapped ion mobility MS.
• Fifty percent more HLA-I peptides with optimized polygon placement, ion accumulation, and CE.
• Up to 15,000 distinct HLA-I and HLA-II peptides from 4e7 cells by single-shot MS/MS.
• High-throughput single-shot analysis identifies >800 HLA-I peptides from 1e6 cells.
• CTA and nuORF HLA peptides detected from as little as 1e6 cells or 15 mg tissue.

## Graphical Abstract

**Figure:**
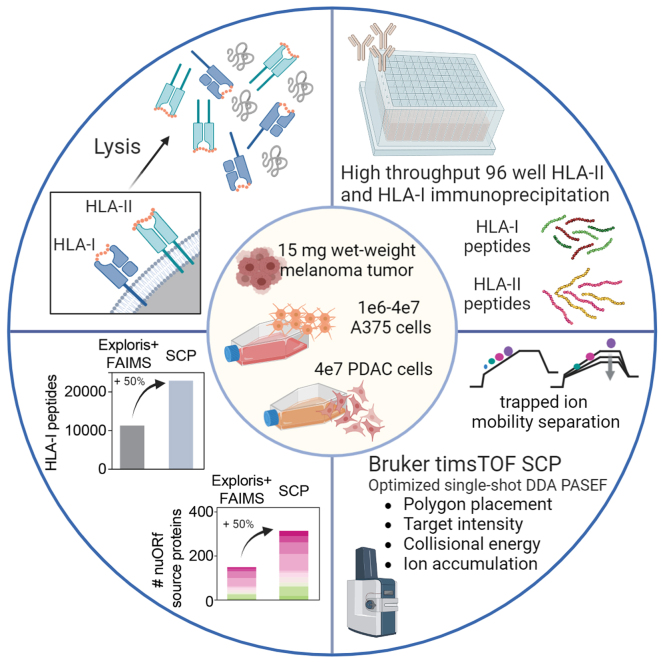

## In Brief

In-depth mass spectrometry–based profiling of human leukocyte antigen (HLA) peptides currently requires large amounts of sample, and off-line fractionation is frequently used to improve coverage. However, primary tissues are often input limited and therefore not amenable to such workflows. Here, we developed and optimized a single-shot acquisition strategy on the timsTOF single-cell proteomics for sensitive and high-throughput immunopeptidome analysis. We demonstrate >2-fold increase in identfied HLA-I peptides and propose multiple collision energy slopes for samples with different HLA-I peptide-binding motifs.

Antitumor CD8+ and CD4+ T cells can identify tumor cells through the recognition of peptide antigens presented by human leukocyte antigen class I (HLA-I) and class II (HLA-II) cell surface molecules, respectively. Immunotherapies that harness the ability of T cells to recognize aberrant cells in the body have shown great clinical potential (1). Thus, personalized cancer vaccines and cell therapies targeting HLA peptides that contain patient-specific somatic mutations (neoantigens) and tumor antigens have shown success in early stage clinical trials (2, 3, 4). In addition, tumor-specific noncanonical antigens, derived from translation of non–protein-coding regions or from noncanonical transcription and/or translation, could have great potential as novel therapeutic targets (5, 6, 7). Therefore, it is crucial to have technologies that enable comprehensive analysis of peptides presented by both HLA-I and HLA-II molecules on tumors.

The highly polymorphic nature of HLA alleles makes their peptide binding rules complex to characterize. Almost all cells in the body express HLA-I molecules that present peptides of length 8 to 12 amino acids, with specific amino acids usually at position 2 and the C-termini of each peptide, so called anchor residues that interact with the HLA-I binding groove (8). In contrast, HLA-II molecules present longer peptides, usually 12 to 25 amino acids in length. HLA-II peptides have allele-specific binding registers that usually contain four anchor residues that can be anywhere within the peptide sequence and are generally expressed by antigen-presenting cells or epithelial cells exposed to interferon gamma (8). Because of these expression patterns and the well-documented cytotoxic activity of CD8+ T cells, HLA-I tumor immunopeptidomes are most frequently studied (1, 5, 8). However, antigen presentation *via* HLA-II to CD4+ T cells plays an essential role in the activation of both B-cell and T-cell immune responses, and several studies have demonstrated that neoantigen-specific CD4+ T-cell–directed therapies can inhibit tumor growth (9, 10, 11, 12, 13, 14, 15). In this context, HLA-II-restricted neoantigens can be presented by antigen-presenting cells that degrade tumor cells through endocytosis and by some HLA-II-expressing tumors (16, 17). Activated neoantigen-specific CD4+ helper T cells can release cytokines and chemokines that enhance antitumor CD8+ cytotoxic T-cell responses (10, 17). In addition, cytotoxic CD4+ T cells can secrete granzyme B and perforin to kill the tumor cells in an HLA-II-restricted manner (18). Therefore, both HLA-I and HLA-II tumor antigen profiling are needed, as the latter play an important role in antitumor immunity in both HLA-II- and non-HLA-II-expressing tumors (9, 10, 11, 12, 13, 14, 15).

Mass spectrometry (MS) has proven to be a powerful technology to define and characterize HLA-I and HLA-II immunopeptidomes. In 1992, Hunt *et al.* (19) sequenced eight HLA-I peptides from 2 billion cells post fractionation using microcapillary high-performance liquid chromatography–electrospray ionization–tandem MS. Current methods and instrumentation for MS-based immunopeptidome analyses now enable thousands of HLA-I and HLA-II peptides to be identified from similar amounts of starting material (20, 21, 22). Yet achieving significant depth (*i.e.*, >10,000 HLA peptides) to identify rare and clinically relevant antigens by LC–MS/MS-based immunopeptidomics from a single HLA immunopurification of <500 million cells remains a challenge (23, 24, 25). More recently, microscaled basic reversed-phase (brp) fractionation of enriched HLA peptides and ion mobility (IM) coupled to LC–MS/MS enabled deep coverage of the HLA peptidome and detection of neoantigen peptides from only 100 million cells or 150 mg wet tumor weight (26). However, reducing sample complexity through offline fractionation increases the number of LC–MS/MS runs per sample threefold to sixfold and is not suitable for sample amounts lower than 100 mg because of accompanying adsorptive peptide losses (26). Current methods therefore are not sufficiently scalable for immunopeptidome profiling of large sample cohorts or primary patient samples where tissue amounts are limited.

With the goal to develop MS-based immunopeptidomics methods with throughput sufficient to analyze hundreds of patient samples while achieving high depth, we investigated single-shot LC–MS/MS complemented by IM separation. Here, we comparatively evaluate single-shot methods on the Thermo Scientific Orbitrap Exploris 480 with field asymmetric waveform ion mobility spectrometry (FAIMS) Pro (Exploris + FAIMS) and the Bruker timsTOF single-cell proteomics (SCP) mass spectrometers. Acquisition methods for the SCP were optimized and evaluated for the analysis of both HLA-I and HLA-II peptides capitalizing on trapped IM mass spectrometry (TIMS) separation and parallel accumulation-serial fragmentation (PASEF) (27, 28, 29). We found that use of the SCP, a dedicated low-input instrument, improved depth of HLA-I and HLA-II immunopeptidomes greater than twofold while increasing throughput nearly fourfold relative to offline brp fractionation. Our improved workflow demonstrates useful immunopeptidome coverage from only 1e6 cells or 15 mg tumor tissue and up to 15,000 distinct HLA-I and HLA-II peptides from 4e7 cells. These optimized single-shot immunopeptidomics methods developed using the SCP instrument have sufficient throughput and reproducibility for profiling the immunopeptidome at scale, thus enabling the analysis of large clinical sample cohorts for the detection of clinically relevant antigens.

## Experimental Procedures

### Culture of A375 Melanoma Cells and 2D Pancreatic Ductal Carcinoma Derived from Primary Human Samples

Conditions for growth and *in vitro* propagation of A375 melanoma were as per American Type Culture Collection guidelines, and conditions for pancreatic ductal adenocarcinoma (PDAC) tumor cell lines were described previously (30). For all cell lines, cells were counted by automated cell counting after trypan blue staining, specified cell amounts ranging from 0.5 to 100 million cells or 1 billion cells for bulk HLA peptide enrichment were harvested by trypsinization (trypsin–EDTA 0.25%; Gibco, catalog no.: 25200056), pelleted, and rinsed in PBS twice. Pellets were either snap frozen or used directly for HLA enrichment.

### Collection of Melanoma Tumor Samples

Melanoma tumor samples were collected as part of the National Institutes of Health/National Cancer Institute Clinical Proteomic Tumor Analysis Consortium (CPTAC) consortium (https://proteomics.cancer.gov/programs/cptac) with protocols mandated by the CPTAC program office. Data collection and analysis in this study was performed in accordance with the Declaration of Helsinki, and Institutional review boards at tissue source sites reviewed protocols and consent documentation adhering to the CPTAC guidelines. Information on participants is not available to the investigators. Samples received by the Broad Institute are fully deidentified, and we do not conduct either human or animal research on site. We have applied and have been approved for the “not engaged status” with the number NE-7303 as designated by the National Institutes of Health.

### HLA-I Peptide Enrichment and Peptide Elution

Cell pellets were lysed in lysis buffer containing 20 mM Tris, pH 8.0, 100 mM NaCl, 6 mM MgCl_2_, 1 mM EDTA, 60 mM octyl β-d-glucopyranoside, 0.2 mM iodoacetamide, 1.5% Triton X-100, 1× Complete Protease Inhibitor Tablet-EDTA free, and 1 mM PMSF in total of 1.2 ml lysate per 50 million cells as described previously (31). Each lysate was transferred to an Eppendorf tube, incubated on ice for 30 min with 2 μl of benzonase (Thomas Scientific; catalog no.: E1014-25KU), and inverted every 5 min. Then lysates were centrifuged at 15,000 rcf for 20 min at 4 °C, and the supernatants were transferred to 96 deep-well plates (Cytiva; catalog no.: 7701-5200).

Immunoprecipitation (IP) of HLA-I only or both HLA-I and II was performed sequentially as follows: ∼37.5 μl prewashed gamma bind sepharose beads (MilliporeSigma; catalog no.: GE17-0886-01) and 15 μg of HLA-II antibody mix (9 μg TAL-1B5 [Abcam; catalog no.: ab20181]), 3 μg EPR11226 (Abcam; catalog no.: ab157210), and 3 μg B-K27 (Abcam; catalog no.: ab47342) were added to lysates in each well, and plates were sealed with 96-well square sealing mats (Thermo Fisher Scientific; catalog no.: AB0675). HLA complexes were captured on the beads by incubating end over end on a rotor at 4 °C for 3 h. Following the incubation beads and lysates were transferred to a prewashed 10 μm PE fritted plate (Agilent; catalog no.: S7898A) stacked on top of a fresh 96 2 ml deep-well plate. Beads with HLA-II peptides complexes were retained on the filter plate, whereas lysates were collected in the fresh 2 ml 96-well plate. For HLA-I enrichment, 37.5 μl prewashed beads and 15 μg of HLA-I antibody (W6/32) (Abcam; catalog no.: 22432 or Novus Biologicals; catalog no.: NB100-64775) were added to the filtered lysates in each well of the 96-well plate and incubated end over end at 4 °C for 3 h.

Beads were transferred and washed on a 10 μm PE fritted plate placed on a positive pressure manifold, as described previously (31). HLA peptides were eluted and desalted from beads using the tC18 40 mg Sep-Pak desalting plate (Waters) as recommended by the manufacturer. The peptides were eluted from the Sep-Pak desalt plate using 250 μl 15% acetonitrile (ACN)/1% formic acid (FA) and 2× 250 μl of 50% ACN/1% FA in 96 deep-well plates, snap frozen, and lyophilized.

Dried peptides were stored at −80 °C until microscaled brp separation on SDB-XC stage tips as previously described (26). Briefly, peptides were reconstituted in 200 μl 3% ACN/5% FA, loaded on equilibrated StageTips with two punches of SDB-XC material (CDS Analytical; previously Empore 3M; catalog no.: 13-110-059). After three washes with 1% FA, HLA-I and HLA-II peptides were eluted with increasing ACN concentrations of 5/10/30% in 0.1% NH_4_OH at pH 10, to 100 μl 96-well PCR plates, frozen, and dried down by vacuum centrifugation.

### LC–MS/MS Analysis

Peptides were reconstituted in 3% ACN/5% FA prior to loading onto an analytical column (35 cm, 1.9 μm C18 [Dr Maisch HPLC GmbH], packed in-house PicoFrit 75 μm inner diameter, 10 μm emitter [New Objective]). For acquisition of the fractionated PDAC samples on the Exploris, peptides were loaded in solvent A (0.1% FA/3% ACN) eluted with a linear gradient (EasyNanoLC 1200; Thermo Fisher Scientific) ranging from 6 to 30% solvent B (0.1% FA/90% ACN) over 84 min, 30 to 90% B over 9 min, and held at 90% B for 5 min at 200 nl/min. Chromatographic separation of Exploris + FAIMS was performed using a linear gradient (Vanquish Neo; Thermo Fisher Scientific) ranging from 2 to 15% solvent B (0.1% FA in 99.9% ACN) over 60 min, 15 to 23% B over 30 min, 23 to 15% B over 10 min, 35 to 80% B over 10 min, and held at 80% B for 10 min at 200 nl/min. MS1 spectra were acquired from 350 to 1700 *m/z* at 60,000 resolution and collected until either 100% normalized automatic gain control target or a maximum injection time of 50 ms was reached. The RF amplitude applied to the RF lens was set to 40%, and advanced peak determination was enabled. Monoisotopic peak detection was set to “peptide,” and “relax restrictions” were enabled. The precursor fit threshold was set to 50% at 1.2 or 1.4 *m/z* fit window for Exploris and Exploris + FAIMS, respectively, with a dynamic exclusion of 10 s at 10 ppm. Potential precursors with charge states +1 to 4 for HLA-I and +2 to 6 for HLA-II above an intensity threshold of 1e4 were isolated in data-dependent acquisition (DDA) mode with a 1.1 *m/z* window, until 50% automatic gain control target or 120 ms maximum injection time was reached. HLA-I precursors were fragmented at 30% normalized higher energy collisional dissociation and HLA-II at 34%, both with 15,000 resolution. When the FAIMS interface was used, spray voltage was increased to 1900 V at “standard resolution.” FAIMS compensation voltages of −50 and −70, each with a cycle time of 1.5 s, were used. Instrument performance was evaluated using an in-house-generated 10 ng tryptic jurkat proteome digest (protein quantification), and data were acquired above a peptide yield threshold of >16,000 peptides for a 120 min LC gradient on the Exploris + FAIMS.

For acquisition on the SCP, peptides were loaded in solvent A (0.1% FA), separated with a linear and stepped gradient with the Bruker nanoElute ranging from 2 to 15 solvent B (0.1% FA in ACN) over 60 min, 15 to 23% in 30 min, 23 to 35% in 10 min, 35 to 80% in 10 min, and held at 80% for 10 min at 400 nl/min. MS1 scans were acquired from 100 to 1700 *m/z* and 1/K0  =  1.7 Vs cm^−2^ to 0.6 Vs cm^−2^ for HLA-I or 1/K0  =  1.3 Vs cm^−2^ to 0.6 Vs cm^−2^ for HLA-II in DDA-PASEF mode. Ten PASEF ramps were acquired with an accumulation and ramp time of 166 ms or as stated otherwise. Precursor above the minimum intensity threshold of 1000 were isolated with 2 Th at < 700 *m/z* or 3 Th >800 *m/z* and resequenced until a target intensity of 10,000 considering a dynamic exclusion of 40 s or as stated otherwise. The collision energy (CE) was lowered linearly as a function of increasing mobility starting from 55 eV at 1/K0  =  1.6 Vs cm^−2^ to 10 eV at 1/K0  =  0.6 V cm^−2^ or as specified. Standard polygon placement was adapted for singly charged HLA-I peptide species and as detailed in the Results section. Instrument performance was evaluated using an in-house-generated 50 ng tryptic jurkat proteome digest (protein quantification), and data were acquired above a peptide yield threshold of >85,000 peptides for a 120 min LC gradient on the SCP.

### HLA Peptide Search and Identification

Mass spectra were interpreted using the Spectrum Mill (SM) software package, version 8.01 (Broad Institute; proteomics.broadinstitute.org) as previously described (26, 32, 33) with modifications detailed later. Briefly, only MS/MS spectra with precursor sequence MH+ in the range 700 to 2000 for HLA-I and 700 to 4000 for HLA-II, a precursor charge 1 to 4 for HLA-I and 2 to 6 for HLA-II, or a minimum of <5 detected peaks were extracted. Merging of similar spectra with the same precursor *m/z* acquired in the same chromatographic peak was enabled. For Exploris, merging was limited to spectra with precursor selection purity of greater than 75%. Precursor selection purity calculates the proportion of ion current in the isolation window of a high-resolution MS1 scan represented by the isotope cluster of precursor ions assigned to the resulting MS/MS scan. MS/MS spectra with a sequence tag length >1 (*i.e.*, minimum of three masses separated by the in-chain masses of two amino acids) were searched with no-enzyme specificity; instrument: ESI-QEXACTIVEHCD-HLA-v3; fixed modifications: carbamidomethylation of cysteine; variable modifications: oxidation of methionine, pyroglutamic acid at peptide N-terminal glutamine, cysteinylation, protein N-terminal acetylation and deamidation; precursor mass tolerance of ±10 ppm; product mass tolerance of ±10 ppm for Exploris or ±15 ppm for SCP data; and minimum matched peak intensity (percent scored peak intensity or %SPI) of 40%. SPI is the percent of product ion intensity (after peak detection) that is matched to a scored ion type.

MS/MS spectra were searched against a compiled database comprised of the human reference proteome Gencode 34 (HLA-I) or 42 (HLA-II) (ftp.ebi.ac.uk/pub/databases/gencode/Gencode_human/release_34/42) with 47,429 or 50,872 nonredundant protein-coding transcript biotypes mapped to the human reference genome GRCh38, 602 common laboratory contaminants, 2043 curated small ORFs (lncRNA and upstream ORFs [uORFs]), 237,427 novel unannotated ORFs (nuORFs) supported by ribosomal profiling nuORF DB v1.037 for a total of 287,501 or 290,944 entries, respectively (6). All databases used in this study are deposited alongside the raw data.

Peptide spectrum matches (PSMs) within <1% false discovery rate (%FDR) using the target decoy estimation of the SM autovalidation module were filtered for a sequence length of 8 to 12 amino acids or 12 to 40, a minimum backbone cleavage score (BCS) of 5 or 7 for HLA-I or HLA-II peptides, respectively. BCS is a peptide sequence coverage metric to enforce a uniformly higher minimum sequence coverage for each PSM. The BCS is a sum after assigning a 1 or 0 between each pair of adjacent amino acids in the sequence (maximum score is peptide length −1) considering all selected ion types to decrease false-positive spectra having fragmentation in a limited portion of the peptide by multiple ion types.

PSMs were consolidated to peptides using the SM protein/peptide summary module case-sensitive peptide-distinct mode. A distinct peptide was the single highest scoring PSM of a peptide detected for each sample. Different modification states observed for a peptide were each reported when containing amino acids configured to allow variable modifications; a lowercase letter indicates the variable modification (C-cysteinylated and c-carbamidomethylated). In addition, precursor fragmentation was evaluated through the percent-dissociated intensity (PDI). The PDI reports the intensity of residual precursor and its neutral losses of water and ammonia subtracted from the total peak intensity in the MS/MS spectrum divided by the total peak intensity.

For MS1 quantification of both SCP and Exploris + FAIMS, we used MSFragger 3.6 (https://msfragger.nesvilab.org/) (34, 35) within FragPipe 19.0 and IonQuant 1.8.9 (36) and Philosopher 4.7.0 (37) to search spectra against the Ensembl v100 (March 2020) database including 71,704 nonredundant protein-coding transcript biotypes mapped to the human reference genome GRCh38, 602 common laboratory contaminants, 2043 curated small ORFs (lncRNA and uORFs), 237,427 nuORFs supported by ribosomal profiling nuORF DB v1.553 for a total of 311,776 entries and 50% reverse peptide sequences. For this, we used a precursor mass tolerance of ±20 ppm for the SCP or ±15 ppm for the Exploris and a fragment mass tolerance of 10 ppm including minimum five matched fragment ions. We performed nonspecific enzymatic protein digestion within 7 to 14 amino acids per peptide in a mass range of 600 to 3000 and a precursor charge 1 to 4. A maximum of three variable modifications per peptides were allowed including oxidation of methionine, N-terminal acetylation and deamidation, cysteinylation, pyroglutamic acid at peptide N-terminal glutamine, and fixed cysteine carbamidomethylation. For IonQuant, we used 10 ppm mass tolerance, 0.05 IM tolerance, 0.4 RT tolerance at a 1% ion, peptide, and protein FDR without match between runs. Data were filtered for 1% FDR on PSM and peptide level and subsequently filtered for peptides common to SM peptideExport.

Identified peptides were filtered for common contaminants, peptides for which both the preceding and C-terminal amino acids were tryptic residues and peptides observed in negative control runs as described previously (32, 33). Subsequent data analysis and visualization was performed in the R computational environment using ggplot2 and UpSetR packages (38, 39).

### Subset-Specific FDR Filtering for nuORFs

While the aggregate FDR for was set to <1%, as described previously, FDR for subset of nuORFs (<5% of total of HLA-I peptides) required more stringent score thresholding to reach a suitable subset-specific FDR <1.0%. To this end, we devised and applied subset-specific filtering approaches. Subsets of nuORF types were thresholded independently in the HLA datasets using a two-step approach. First, PSM scoring metric thresholds were tightened in a fixed manner for all nuORF PSMs so that nuORF distributions for each metric improved to meet or exceed the aggregate distributions. For all ‘omes, the fixed thresholds were minimum score: 7, minimum %SPI: 50%, precursor mass error: ±5 ppm, minimum BCS: 5, and sequence length: 8 to 12 (HLA-I) and 9 to 50 (HLA-II). Second, individual nuORF type subsets with FDR estimates remaining above 1% were further subject to a grid search to determine the lowest values of BCS (sequence coverage metric) and score (fragment ion assignment metric) that improved FDR to <1% for each ORF type in the dataset.

### HLA Peptide Prediction

HLA peptide prediction was performed using HLAthena (hlathena.tools) (33). Unless otherwise specified, peptides were assigned to an allele using a percentile rank cutoff of ≤0.5.

### Experimental Design and Statistical Rationale

**Fig. 1:**
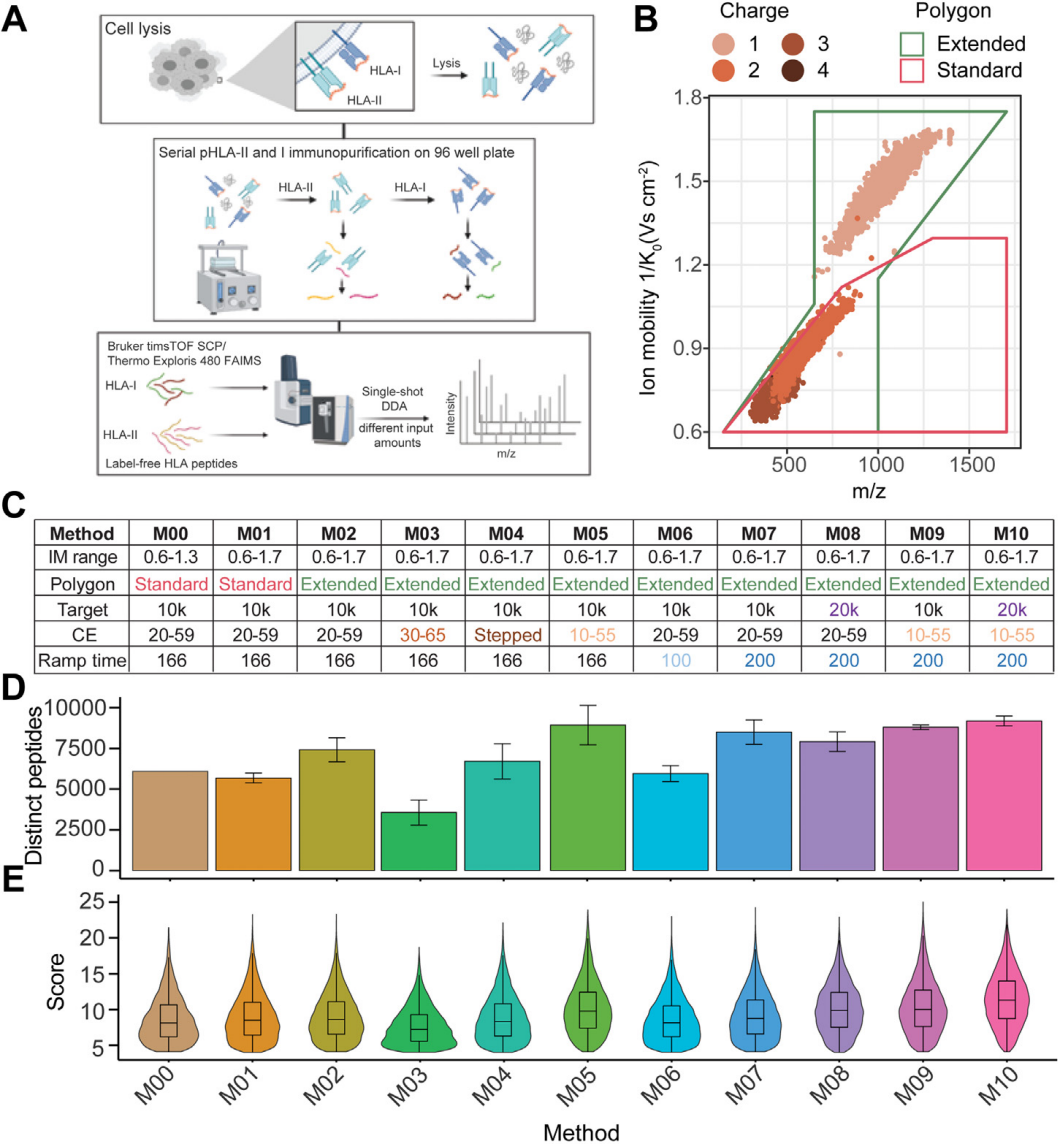
**Human leukocyte antigen (HLA) sample preparation overview and timsTOF single-cell proteomics (SCP) acquisition method optimization for analysis of HLA-I peptides enables inclusion of singly charged peptides.***A*, schematic overview of HLA sample preparation and mass spectrometry (MS) acquisition schemes. Serial HLA-I and HLA-II enrichment, acid elution of peptides from HLA complexes, and peptide desalting performed in a semiautomated 96-well format, followed by single-shot data-dependent acquisition (DDA) analysis of purified HLA-I and HLA-II peptides using our standard workflow with the Exploris + FAIMS or the timsTOF SCP. *B*, HLA-I peptide spectrum matches (PSMs) identified on the timsTOF SCP using the standard precursor filter (“*polygon*”) in *pink* and the extended polygon in *green* across *m/z* and ion mobility (IM) dimensions. Colors indicate precursor charge state. *C*, timsTOF SCP parameter overview, including IM range, polygon placement, target intensity value (“target”), collision energy (CE) slope, accumulation, and ramp time for M00–M10. *D*, number of unique HLA-I peptides from 1e7 cell equivalents from bulk sample preparation postfiltering in duplicates with M00–M10 on the timsTOF SCP. Median and standard deviation are shown. *E*, corresponding score distributions of HLA-I peptides for methods indicated in *D*. FAIMS, field asymmetric waveform ion mobility spectrometry.

**Fig. 2:**
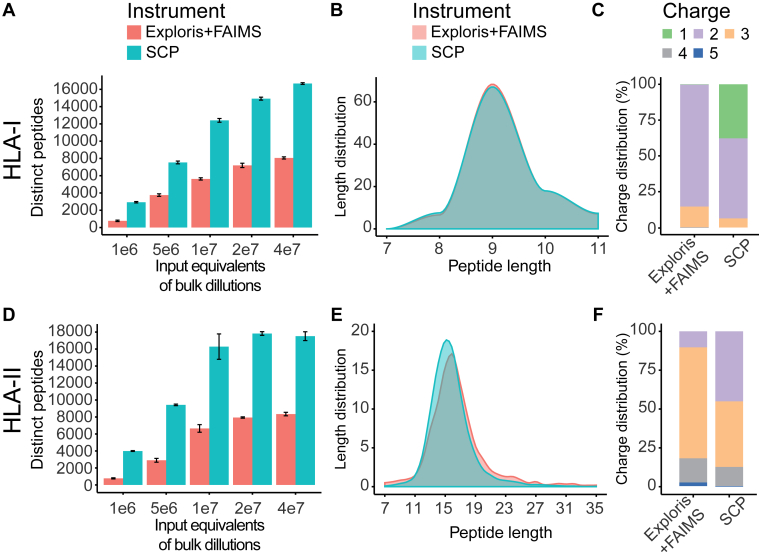
**Single-shot acquisition of HLA-I and HLA-II peptides on the timsTOF single-cell proteomics (SCP) increases identifications greater than twofold compared with Exploris + FAIMS.***A*, unique HLA-I peptides identified by single injections on Exploris + FAIMS (*red*) and timsTOF SCP (*blue*) from bulk digest at indicated input equivalents. Mean and standard deviation is shown. *B*, peptide length distribution across HLA-I peptides identified on the Exploris + FAIMS (*red*) and the timsTOF SCP (*blue*) from bulk digest at indicated input equivalents from bulk sample preparation. *C*, HLA-I peptide charge states for Exploris + FAIMS or timsTOF SCP. *D*, unique HLA-II peptides identified by single injections on Exploris + FAIMS (*red*) and timsTOF SCP and indicated input equivalents (*blue*) from bulk sample preparation. Mean and standard deviation is shown. *E*, HLA-II peptide length distribution for Exploris + FAIMS (*red*) and the timsTOF SCP (*blue*). *F*, charge state distribution for HLA-II peptides on Exploris + FAIMS or timsTOF SCP. FAIMS, field asymmetric waveform ion mobility spectrometry; HLA, human leukocyte antigen.

**Fig. 3:**
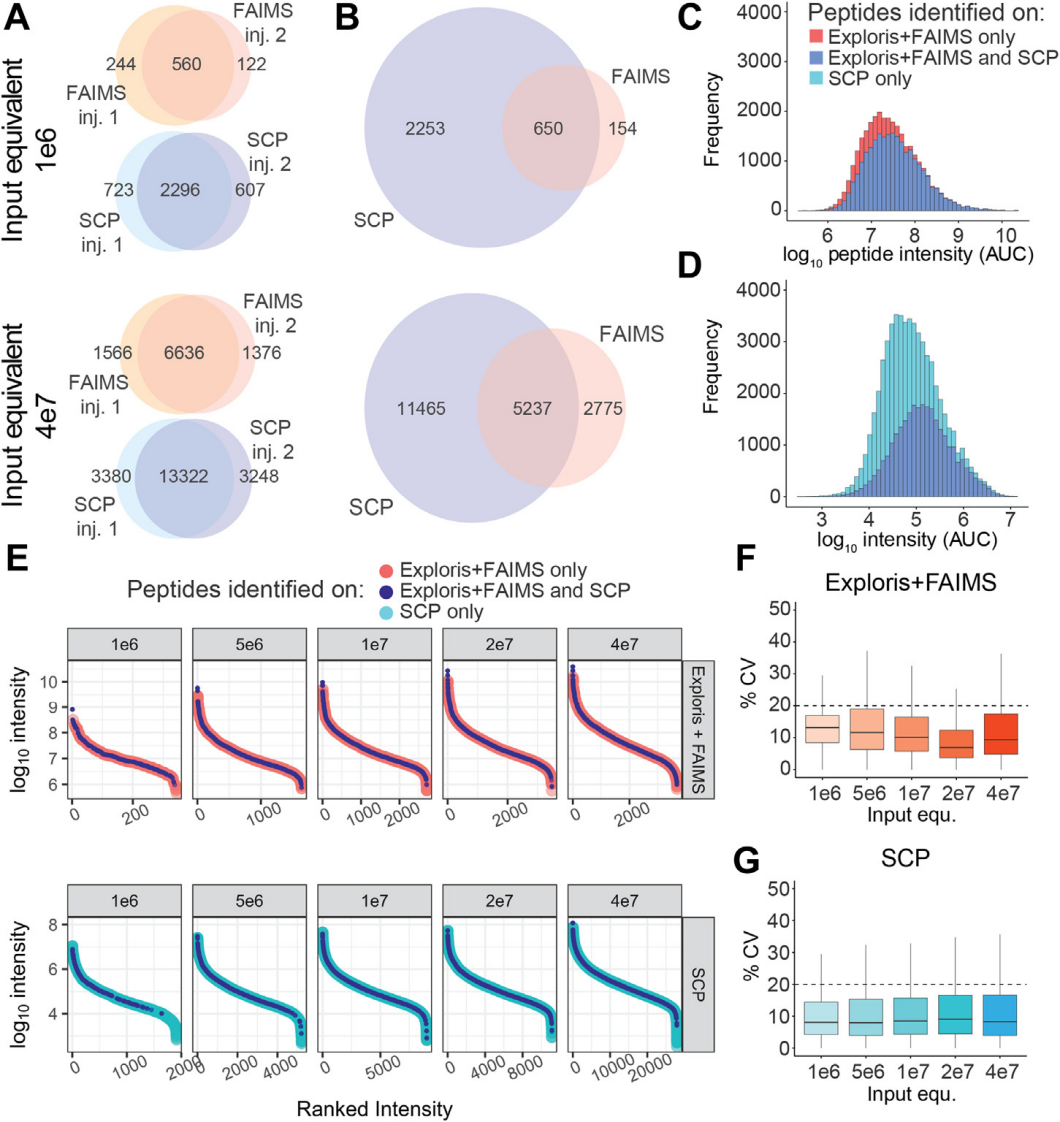
**HLA-I peptide identifications on the timsTOF single-cell proteomics (SCP) show high reproducibility and extended dynamic range.***A*, overlap of HLA-I peptides across multiple injections on the Exploris + FAIMS or the timsTOF SCP at 1e6 or 4e7 A375 cell input equivalents from bulk sample preparation. *B*, overlap of HLA-I peptides between the Exploris + FAIMS and the timsTOF SCP at 1e6 (*upper panel*) or 4e7 (*lower panel*) A375 cells. *C*, Log_10_ intensity (MS1 AUC) distribution of HLA-I peptides uniquely identified on the Exploris + FAIMS (*pink*) or both instruments (*dark blue*). *D*, Log_10_ intensity (MS1 AUC) distribution of HLA-I peptides uniquely identified on the timsTOF SCP (*light blue*) or both instruments (*dark blue*). *E*, ranked peptide intensity at different cell input equivalents from bulk sample preparation on the Exploris + FAIMS (*upper panel*) or the timsTOF SCP (*lower panel*). Respective intensity of peptides uniquely identified on the Exploris + FAIMS (*pink*), the timsTOF SCP (*light blue*), or both instruments (*dark blue*). *F*, percent CV of peptide intensity between technical replicates at different cell input equivalents from bulk sample preparation on Exploris + FAIMS. *Dashed line* indicates 20% CV. *G*, % CV of peptide intensity between technical replicates identified on the timsTOF SCP at indicated input equivalents from bulk sample preparation. *Dashed line* indicates 20% CV. AUC, area under the curve; FAIMS, field asymmetric waveform ion mobility spectrometry; HLA, human leukocyte antigen; MS, mass spectrometry.

**Fig. 4:**
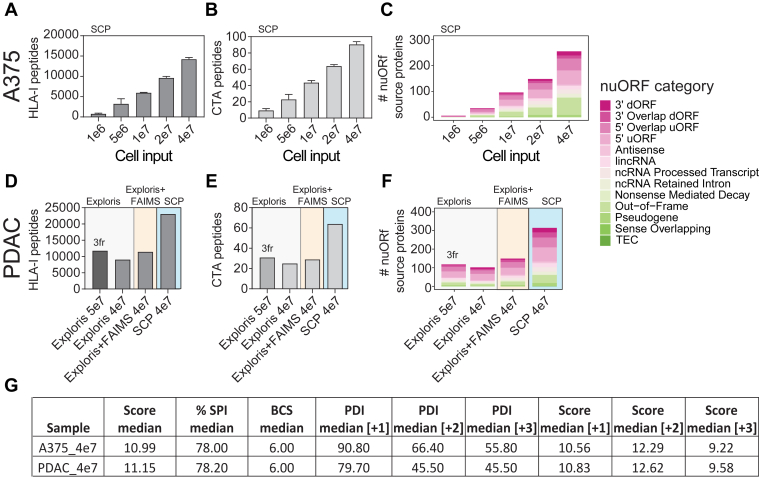
**Single-shot acquisition of HLA-I peptides from low amounts of tumor-derived cell lines enables detection of cancer-testis antigen (CTA) and novel unannotated ORFs (nuORFs)–derived peptides on the timsTOF single-cell proteomics (SCP).***A*, unique HLA-I peptides directly enriched from 1 to 40 million A375 cells as indicated by single-shot injections on timsTOF SCP. *B*, peptides detected in *A* that map to CTA source proteins from CTdatabase (44). *C*, unique nuORF source proteins contributing to HLA-I immunopeptidome of 1 to 40 million A375 cells (6). *D*, unique HLA-I peptides identified from 40 to 50 million pancreatic ductal adenocarcinoma (PDAC) line in three offline StageTip fractions on the Exploris (Exploris, 3fr) or single-shot injections on Exploris ± FAIMS or the timsTOF SCP. *E*, peptides detected in *D* that map to CTA source proteins from CTdatabase (44). *F*, unique nuORF source proteins identified in a patient-derived PDAC cell line with the acquisition schemes indicated in *D*. *G*, quality metrics for HLA-I peptides from 4e7 A375 or PDAC samples calculated by Spectrum Mill including median score, %SPI, BCS, and median PDI by charge state on the SCP. %SPI, percent scored peak intensity; BCS, backbone cleavage score; HLA, human leukocyte antigen; PDAC, pancreatic ductal adenocarcinoma; PDI, percent precursor dissociation intensity.

**Fig. 5:**
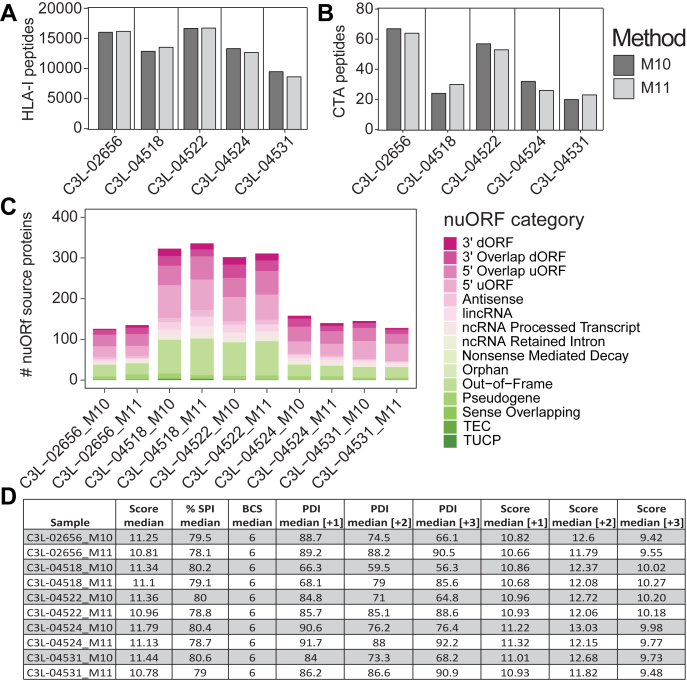
**Single-shot DDA-PASEF of low-input primary melanoma tumors on the timsTOF single-cell proteomics (SCP) enables deep profiling of the HLA-I immunopeptidome and detection of cancer-testis antigen (CTA) and novel unannotated ORF (nuORF)–derived peptides.***A*, unique HLA-I peptides enriched from 15 mg wet weight primary melanoma tumors (estimated <1e7 cells) identified by single-shot injections on timsTOF SCP with methods M10–M11. *B*, peptides detected in *A* that map CTA source proteins from CTdatabase (44). *C*, unique nuORF source proteins represented in HLA-I immunopeptidome of the respective melanoma tumors (6). *D*, quality metrics of HLA-I peptides from primary melanoma tumors including median score, %SPI, BCS, median PDI, and scores by charge state using M10 or M11 on the SCP. %SPI, percent scored peak intensity; BCS, backbone cleavage score; DDA, data-dependent acquisition; HLA, human leukocyte antigen; PASEF, parallel accumulation-serial fragmentation; PDI, percent precursor dissociation intensity.

Sample preparation and data acquisition parameters for both instruments are detailed in the aforementioned sections. HLA peptides were enriched in bulk from 1e9 A375 cells, and aliquots corresponding to 1e7 cells were injected in technical duplicate for method optimization on the SCP (Fig. 1; n = 2). Titration experiments using 1e6 to 4e7 cell aliquots of the bulk purified HLA-I peptides were analyzed in technical triplicate (Figs. 2 and 3; n = 3). Direct IP experiments were carried out by enriching HLA peptides from starting amounts of cells corresponding to 1e6 to 4e7 million cells in triplicates (Fig. 4; n = 3). About ∼15 mg wet weight of primary melanoma tumors were used for HLA-I peptide enrichment from five patients in single-shot injections because of tissue constraints (Fig. 5; n = 5). Sample size was n = 1 for PDAC line and primary melanoma tumor samples per instrument setup because of sample input limitations. Median peptide counts across replicates ± standard deviation and Pearson correlation of peptide intensities are shown.

## Results

### Single-Shot HLA-I and HLA-II Method Optimization and Benchmarking

To improve depth, sensitivity, and throughput of our existing immunopeptidomics analysis workflow, we focused on optimizing MS acquisition and directly compared single-shot LC–MS/MS DDA acquisition on the SCP to a recently published method using the Exploris + FAIMS (26, 31). We aimed for a single-shot LC–MS/MS analysis to minimize sample handling related to biochemical fractionation and to enable high-throughput analyses of input limited samples. For our initial evaluation, we used 1e7 cell equivalents of HLA-I and HLA-II peptides derived from a bulk preparation of 1e9 A375 cells, a multiallelic melanoma cell line (Fig. 1*A*). A375 expresses lower levels of TAP genes and has moderate HLA-I and HLA-II expression, making it well suited for optimizing serial HLA immunopeptidome analysis (40). A recent report described MS-based immunopeptidome analysis of 4e8 A375 cells in which ∼10,000 HLA-I peptides were identified using a Thermo Scientific Orbitrap Eclipse (41). To achieve maximum immunopeptidome depth at high throughput on the SCP, we investigated a wide range of precursor selection, accumulation time, and fragmentation parameters to identify the optimal combination for HLA alleles expressed by A375 (Fig. 1, *B* and *C* and supplemental Table S1).

In the dual-TIMS analyzer within the SCP, ions are separated by their collisional cross-section (CCS), based on their size, shape, and charge. This allows filtering of the selection of potential precursors for MS/MS based on their IM and *m/z* (42, 43). The “standard” polygon-shaped filter shown in Figure 1*B* (*pink box*) is designed to isolate multiply charged precursors and exclude singly charged contaminants in methods optimized for the analysis of tryptic peptides. Tryptic and HLA-II peptides are more similar in length and charge properties to tryptic peptides than HLA-I peptides are. In contrast, HLA-I peptides are shorter, generally 8 to 12 amino acids long, and allele-specific binding motifs that frequently lack basic amino acids, resulting in singly charged precursors with atypical CCS and IM characteristics. To better capture HLA-I peptides, we first expanded the IM range from standard 1/K0 1.3 Vs cm^−2^ to 1/K0 = 1.7 Vs cm^−2^ (M00–M01; Fig. 1*C*). The extension of the “standard” tryptic polygon resulted in comparable numbers of HLA-I peptides identified relative to M00 (Fig. 1, *D*–*E*). We therefore modified the polygon placement to include singly charged precursors with *m/z* >600 (Fig. 1, *B* and *C*, *green box* and “extended polygon” in M02–M10) and observed a 20% increase in peptide IDs (Fig. 1*D* and supplemental Fig. S1, *A* and *B*). Upon inclusion of singly charged precursors using the extended polygon within M02, we observed that these precursors had a lower PDI distribution compared to higher charged ones (supplemental Fig. S2*B*). As CE on the timsTOF is ramped linearly as a function of IM, we speculated that previously optimized CEs for tryptic peptides are not directly transferable to HLA-I peptides with atypical CCSs, particularly singly charged ones. We tested whether increasing precursor fragmentation could improve identification and linearly increased CEs (30 eV at 1/K0  =  0.6 Vs cm^−2^ to 65 eV at 1/K0  =  1.6 V cm^−2^) relative to M02 (M02 *versus* M03; Fig. 1*C* and supplemental Fig. S2*A*). As expected, this slightly increased median PDI by 2%, 4%, and 2% for +1, +2, and +3 charged precursors, respectively, but surprisingly, this change lowered peptide identifications by over 50% (M02 *versus* M03; Fig. 1*D*; supplemental Figs. S1, *A* and *B* and S2*B*). Therefore, we instead empirically optimized CEs for individual charge states separated by their IM. To accomplish this, we introduced a step to the CE slope, starting with lower CEs at lower IM compared with M02 (1/K0 = 0.6–1.1 V cm^−2^) and a higher CE slope at high IM of method M03 (*i.e.*, 1/K0 = 1.1–1.6 V cm^−2^ for singly charged precursors; supplemental Fig. S2*A*). These changes did not result in the expected peptide ID increase, but to the contrary, decreased IDs by 9.5% (M02 *versus* M04; Fig. 1*D* and supplemental Fig. S1, *A* and *B*). We then tested lowering the CE slope (10 eV at 1/K  =  0.6 Vs cm^−2^ to 55 eV at 1/K0  =  1.6 Vs cm^−2^). This change decreased median PDI by 5%, 34%, and 46% for +1, +2, and +3 charged precursors, respectively (M02 *versus* M05; supplemental Fig. S1, *B* and *E*), but unexpectedly, peptide IDs increased by 16% and the %SPI increased by 5% (M02 *versus* M05; Fig. 1*C* and supplemental Fig. S1, *A*–*C*).

Next, to increase scanning speed and thus decrease cycle time, we reduced the standard TIMS ramp time from 166 to 100 ms (M02 *versus* M06; Fig. 1C). The TIMS ramp time defines the initial accumulation time of ions in the first TIMS funnel and the subsequent elution time from the second TIMS to the quadrupole for precursor isolation. However, this change decreased peptide identifications by 15% (Fig. 1, *D*–*E* and supplemental Fig. S1, *A* and *B*). We next evaluated the effect of increasing the ramp time to 200 ms. Increasing the ion accumulation increases the peptide ions available for fragmentation and thus improves HLA-I peptide identifications by 14% (M02 *versus* M07; Fig. 1, *C*–*E* and supplemental Fig. S1, *A* and *B*). Doubling the target intensity from 10,000 to 20,000 improved the median peptide score by 19% and marginally increased peptide identifications by 5%, presumably by improving the coverage of complementary b- and y-type ions (M02 *versus* M08; Fig. 1, *C*–*E* and supplemental Fig. S1, *A*–*E*). Based on the collective results obtained from our testing, we designed method M10 that combines optimized polygon filter placement, lower CE slope of 10 eV at 1/K0  =  0.6 Vs cm^-^^2^ to 55 eV at 1/K0  =  1.6 Vs cm^−2^, 200 ms ramp time, and a higher target intensity threshold of 20,000 (Fig. 1, *C*–*E* and supplemental Fig. S1, *A*–*E*). M10 yielded the highest number of IDs and median scores, we therefore considered parameter set M10 as optimal for HLA-I immunopeptidomics of A375 on the SCP.

### Improved coverage of HLA-I and HLA-II immunopeptidomes on the SCP

With the optimized settings described previously, we next compared single-shot analyses on the SCP to the Exploris + FAIMS. For this, HLA-I and HLA-II peptides were bulk enriched from 1e9 A375 cells, and equivalents of 1e6, 5e6, 1e7, 2e7, and 4e7 cells were injected in triplicates on both instruments (supplemental Tables S2, S3, S5 and S6). Even though we aim to minimize variability between setups, we used two different LC systems because of software incompatibility for which we employed the respective optimal flow rate spray stability as described in the Experimental procedures section (*i.e.*, 200 and 400 nL for Exploris + FAIMS and SCP, respectively). For HLA-I peptides, we observed a twofold increase in identifications using the SCP in comparison to the Exploris + FAIMS at 5e6 cells and higher (Fig. 2*A*). At 4e7 input level, we observed over 17,000 HLA-I peptides on the SCP *versus* ca. 8000 on the Exploris + FAIMS. At 1e6 cells, the optimized SCP method yielded ∼3000 HLA peptides in comparison to ∼800 peptides on the Exploris + FAIMS, a differential of nearly fourfold (Fig. 2*A*). The increase in peptide IDs corresponded to a 1.5-fold increase in the number of source proteins represented in the HLA-I immunopeptidome (supplemental Fig. S3*A*). Based on their length or charge distributions (Fig. 2, *B* and *C*) and the presence of expected anchor residues for A375 alleles (supplemental Fig. S6*E*), we confirmed that the additional peptides identified by the SCP are indeed HLA-I bound. Parameter set M10 on the SCP yielded 92%, 70%, and 64% median PDI for +1, +2, and +3 charged precursors, respectively. While the median PDI using the SCP was lower by 28% and 35% for +2 and +3 charged precursors than on the Exploris + FAIMS, the scores and sequence coverage were comparable (supplemental Fig. S4, *A*–*C*). As expected, more singly charged HLA-I peptides were observed using the SCP than the Exploris + FAIMS as those are generally excluded by the FAIMS compensation voltages used (Fig. 2*C*). Interestingly, spectra on the Exploris + FAIMS are composed predominantly of y-type and internal ions, whereas the SCP yielded more complementary b- and y-type ion pairs, with few internal ions (supplemental Fig. S6*D*). Thus, the SCP enables single-shot HLA-I immunopeptidome profiling using input amounts of 1e6 cells and higher because of the twofold higher peptide IDs at comparable scores and more complementary b- and y-type ion pairs compared with the Exploris + FAIMS (Fig. 2*A* and supplemental Fig. S3*C*).

As HLA-II peptides are longer than HLA-I peptides and present with higher charge states more similar to tryptic peptides (*i.e.*, +2 to +5), we used a previously published method based on default tryptic acquisition methods on the SCP with standard polygon filter, CE 20 eV at 1/K0  =  0.6 Vs  cm^−^2 to 59 eV at 1/K0  =  1.6 Vs cm^−2^ and target intensity threshold of 20,000 (28). We observed a twofold to threefold increase of HLA-II peptide identifications on the SCP compared with the Exploris + FAIMS (Fig. 2*D*). HLA-II peptide length (12–25 amino acids) and charge distribution +2 to +5 were identified on the SCP as expected, with a slight shift in the median length (15 *versus* 16, Fig. 2, *E* and *F*). Moreover, we observed more peptides with charge state +2 on the SCP, whereas peptides identified on the Exploris + FAIMS are predominantly of higher charge (*i.e.*, +3 to +5, Fig. 2*F*). We hypothesize that the Exploris + FAIMS is identifying longer and more highly charged peptides because it leverages a higher charge state–specific CE and likely results in improved fragmentation for longer and high-charge peptides. While the overall number of HLA-II peptide identifications is twofold higher on the SCP, HLA-II peptides on both instruments show comparable scores and sequence coverage (indicated by BCS) to those observed on the Exploris + FAIMS, with slightly lower precursor dissociation of 24%, 7%, 3%, and 2% for +2, +3, +4, and +5 precursors, respectively (supplemental Fig. S5, *A* and *B*). In summary, across input of 1e6 to 4e7 A375 cells, the SCP increased coverage of HLA-I and HLA-II immunopeptidomes by at least twofold compared with the Exploris + FAIMS.

### Reproducibility, Variance, and Dynamic Range of HLA-I Immunopeptidome Analysis on the SCP

To accurately represent biological conditions and capture differences in low abundant clinically relevant antigens, low stochasticity, high sensitivity, wide dynamic range, and reproducible quantification is critical. Consistent with previous reports, we observed a ∼80% overlap of peptide IDs between technical replicates for samples run on the SCP and the Exploris + FAIMS across all input amounts (26, 33) (Fig. 3*A*, supplemental Tables S2 and S3). Figure 3*B* shows the overlap and uniqueness of HLA-I peptides observed on the instruments. At the 1e6 cell input level, only 5% of the 3057 HLA-I peptides identified are unique to the Exploris + FAIMS, whereas 73% are unique to the SCP (Fig. 3*B*, *top panel*). At 4e7 cell input level, 16% of the 19,477 HLA-I peptides observed are unique to the Exploris + FAIMS, whereas 59% were only detected on the SCP (Fig. 3*B*, *bottom panel*). Interestingly, peptide identification overlap excluding singly charged precursors found on the SCP, as they are also excluded by the Exploris + FAIMS method, remains highly similar across cell input levels (*i.e.*, 50% unique for SCP, 20% unique in Exploris + FAIMS; Fig. 2*A* and supplemental Fig. S6*C*). The majority of overlapping peptides were observed in charge states +2 and +3 with the same charge state on both instruments (supplemental Fig. S6*A*). A small percentage (16%) of peptides were exclusively observed as +1 precursors on the SCP but +2 on the Exploris + FAIMS. Similarly, 12% of peptides were detected in both +1 and +2 charge states on the SCP and only in +2 on the Exploris + FAIMS (supplemental Fig. S5*A*). Of note, around 25% of all HLA-I peptides identified using the SCP are observed in multiple charge states compared with <10% on the Exploris + FAIMS (supplemental Fig. S6*B*). The presence of multiple charge states per peptide provides additional confidence for peptide identification, which is particularly advantageous for immunopeptidome and other profiling applications such as post-translational modifications that rely on single peptides.

We hypothesized that >50% more unique HLA peptides are identified on the SCP because of the instruments’ higher sensitivity and sampling speed (Fig. 3*B*). Indeed, the median log_10_ precursor intensity of peptides uniquely identified on the SCP is lower compared with those observed in common (5.1 *versus* 4.9, Fig. 3, *C* and *D*). This is also illustrated by the extension by two orders of magnitude in overall dynamic range using the SCP compared with the Exploris + FAIMS (Fig. 3*E*). The improved sampling of lower abundant peptides and the corresponding increase in dynamic range was observed at all input amounts on the SCP (Fig. 3*E*). The median percent coefficient of variation on each instrument is <20% (Fig. 3, *F* and *G*), with a Pearson correlation of >0.8 between replicates across all input amounts (supplemental Fig. S7, *A* and *B*). Moreover, the SCP demonstrates highly accurate quantitation relative to the expected fold change across the entire dilution series from 1e6 to 4e7, whereas lower input on the Exploris + FAIMS shows less accurate relative quantitation (supplemental Fig. S7, *C* and *D*).

The results presented thus far were based on serial dilutions of bulk enriched A375 immunopeptidomes. To better understand the capabilities to deeply profile HLA-I peptides from input-limited samples, we directly enriched and analyzed HLA-I peptides from low cell numbers. For this analysis, we used the same input range as the bulk experiments and acquired them with M10 on the SCP (Fig. 4*A* and supplemental Table S4). Direct enrichment from 4e7 cells decreased HLA-I peptide identification by only 12% (*i.e.*, 17,000 from bulk enrichment, 15,000 *via* direct IP). As expected, this decrease was amplified at lower cell input. We identified 700 to 900 HLA-I peptides from 1e6 cells, 2000 to 3000 from 5e6 cells, and 10,000 from 2e7 cells, respectively (Fig. 4*A* and supplemental Table S4). The immunopeptidome depth obtained by enrichment from as little as 1e6 A375 cells with our workflow is sufficient to identify HLA-I peptides derived from cancer-testis antigen (CTA). We observed CTA source protein–derived peptides across all input levels, with ∼9 and ∼90 from 1e6 or 4e7 A375 cells, respectively (Fig. 4*B* and supplemental Table S4) (6, 44). We were particularly interested in CTA-derived peptides, as they are expressed across multiple tumor types and therefore represent putative targets for cancer immunotherapy (45). Moreover, we detected ∼5 unique nuORF-derived peptides from 1e6 A375 cells, with highest relative contribution from out-of-frame ORFs and 5′ uORFs across all sample input levels (Fig. 4*C*). Recent studies have demonstrated that peptides originating from noncanonical proteins are displayed on HLA-I molecules (6, 46, 47, 48, 49). These nuORFs may arise from transcripts currently annotated as nonprotein coding, including the 5′ and 3′ untranslated regions, overlapping yet out-of-frame alternative ORFs in annotated protein-coding genes, long noncoding RNAs, or pseudogenes (6). HLA peptides derived from noncanonical proteins may act as additional sources of tumor antigens and have the potential to expand immunotherapy targets in cancer, especially for tumors with low mutation burdens (6, 46, 47, 48, 49). It is therefore of interest that we identified twice as many nuORF source proteins from 4e7 cells with the SCP compared with the Exploris + FAIMS (Fig. 4*F* and supplemental Table S8).

### Application of Acquisition Methods To Samples with Different HLA-I Allele Compositions

We next evaluated the M10 method (Fig. 1*C*) that we optimized using the A375 cells on a sample with HLA alleles and peptide-binding characteristics distinct from A375. For this, we selected a patient-derived PDAC cell line where we had sufficient input material to evaluate both the Exploris + FAIMS and the SCP. We compared use of the single-shot M10 SCP method with single-shot injections ± FAIMS or brp fractionation (3 fr) without FAIMS on the Exploris (supplemental Table S8). Single-shot acquisition from 4e7 PDAC-derived cells on the Exploris yielded 9080 HLA-I peptides without FAIMS and 11,460 with FAIMS. In contrast, 23,000 HLA-I peptides were identified on the SCP from 4e7 PDAC-derived cells, an increase of greater than twofold (Fig. 4*D*). Use of off-line biochemical fractionation into 3 frs and single-shot analysis using the Exploris only marginally increased the numbers of peptides identified *versus* single-shot injections (11,810 *versus* 11,460) while increasing manual sample handling and threefold increased data acquisition time, a trade-off that is impractical for large sample cohorts. Similarly, and consistent with observations in A375 cells, the SCP identifies twofold more CTA and nuORF-derived HLA-I peptides (Fig. 4, *E* and *F*, supplemental Tables S7 and S8).

While the median score, %SPI, and BCS were comparable between the data collected on the Exploris and the SCP for HLA-I peptides derived from the PDAC cells, we noted a >10% decrease in median PDI per charge state when compared with the A375 results (Fig. 4*G* and supplemental Fig. S7*A*). Specifically, PDI for +1, +2, and +3 charged precursors were reduced by 12%, 31%, and 18%, respectively, with +2 and +3 charge states affected the most (Fig. 4*G* and supplemental Fig. S8, *A* and *B*). We concluded that the lower PDI on the SCP compared with Exploris + FAIMS (Fig. 4*G*) was likely because of too low CE for optimal fragmentation of peptides with the HLA allele–specific peptide-binding characteristics present in the PDAC cell line.

Aiming to identify an acquisition method on the SCP that could be universally applied to analyze patient cohorts with diverse HLA alleles, we revisited the CE parameters. Based on the observation that M10 particularly underfragmented multiply charged peptides, we increased the CE at low IM 1/K0  =  0.6 Vs cm^−2^ from 10 eV in method M10 to 15 eV, which we refer to as M11. This marginal increase in CE at low IM yielded comparable numbers of HLA-I peptides to the M10 method but improved PDI for +2 and +3 charged precursors in melanoma samples (supplemental Figs. S4*B* and S8, *A*–*D*). We then tested both the M10 and M11 methods for analysis of the immunopeptidomes of primary melanoma tumors (n = 5) from caucasian patients in another multiallelic set of samples. The higher CE slope of M11 again increased PDI by 0.5 to 2.6% for +1, 13 to 25% for +2, and 17 to 35% for +3 precursors relative to M10 across all samples (supplemental Fig. S9*E*). Importantly, the motifs of peptides identified with M10 and M11 revealed the same anchor residues (supplemental Fig. S10*A*). Overall, we observed comparable numbers of peptides (8847–16,385) and scores (11.77 ± 5%) using M10 and M11 methods starting with just 15 mg cryopulverized tissue (Fig. 5, *A* and *D* and supplemental Fig. S8, *D* and *E*). Moreover, we identified similar numbers of 20 to 60 CTA-derived peptides and 100 to 300 unique nuORF peptides with both methods (Fig. 5, *B* and *C*; supplemental Tables S9 and S10).

We next evaluated peptides exclusively identified by either M10 or M11 and observed an increase in singly charged precursors in M10. The slightly increased CE in M11 yielded more triply charged precursors with a slight shift in length toward 10 to 11 amino acids as compared with 9 amino acids in M10 (supplemental Fig. S10, *B* and *C*). We therefore speculate that higher CE yields longer (>9 amino acids) and higher charged peptides because of improved fragmentation of these peptides.

In conclusion, we find that multiple CE slopes, similar to those presented here, should be tested based on the anticipated sample or cohort-specific HLA-I alleles and their peptide-binding motifs, and we present M11 as our optimal SCP method for HLA-I immunopeptidome profiling of Caucasian populations.

## Discussion

One method that is frequently used to achieve deep immunopeptidome depth to enable detection of therapeutically relevant HLA antigens is offline orthogonal chromatographic separation. However, clinical sample availability is often limited, and offline fractionation does not offer the same benefits at lower input amounts and also significantly decreases sample throughput. To simultaneously improve immunopeptidome depth and increase analysis throughput for low-level samples, we evaluated and optimized acquisition parameters on the Bruker timsTOF SCP for HLA-I and HLA-II peptides. Through systematic evaluation of instrument parameters such as IM precursor selection polygon filter, CE settings, TIMS accumulation, and ramp times and target intensity thresholds, we were able to optimize instrument parameters that greatly improved immunopeptidome depth. At higher input levels of 1e7 cells, we observe ∼10,000 HLA-I or 9000 HLA-II peptides that include peptides derived from CTA and nuORF source proteins. Most importantly, method optimization enabled single-shot immunopeptidome depth of up to 1000 HLA-I peptides from a direct IP of only 1e6 A375 cells and enhanced spectral quality as measured by score. The ability to characterize such low input samples also allows the direct detection of potential therapeutically relevant HLA peptides and facilitates immunopeptidome data collection from smaller and more difficult to obtain tumor biopsies.

Our systematic analysis found that multiple instrument method parameters contributed to the improved immunopeptidome depth. Specifically, we noted that extending the polygon filter placement to include singly charged precursors and decreasing the CE values had the largest impact on total peptide identifications (*i.e.* 35% increase compared to the default tryptic method, Fig. 1*D*). The inclusion of singly charged precursors through adjustment of the polygon filter increased the number of charge states per HLA-I peptide by 15% thereby increasing the identification confidence through detection and fragmentation of the same peptide in multiple charge states. Surprisingly, a lower CE resulted in increased numbers of b- and y-ion pairs, which in turn increased the identification score even though the peptides appear to be relatively under fragmented by their PDI. While decreasing the CE did not impact the BCS, we observed different fragment ion type distributions produced on the Exploris *versus* SCP. Specifically, we identified fewer internal fragment ions on the SCP compared to the Exploris + FAIMS and largely increased b- and b/y fragment ion pairs, contributing to increased scores. We speculate that the high sensitivity of the SCP enables increased detection of the low abundant b/y fragment ion pairs formed as a result of decreased CE. Although the decreased CE slopes improve HLA-I peptide identifications and scores, we hypothesize that implementing precursor fragmentation in a charge state specific manner on the SCP, which is not currently possible with the version of instrument control software used in this study, will further improve peptide fragmentation to improve sequence coverage.

We also applied our optimized SCP analysis methods to profile the immunopeptidomes of a patient-derived PDAC cell line and primary melanoma tumors identifying ∼22,000 HLA-I peptides from 4e7 PDAC cells and 8847 to 16,3885 from 15 mg melanoma tissue. Interestingly, we noticed substantial differences in PDI between the initial method optimization (*i.e.*, A375 cell line) and the subsequent application to patient-derived PDAC cell lines. Despite up to a 50% difference in PDI for multiply charged precursors, we observed comparable overall peptide scores, %SPI, or the percent of peptide backbone cleavage in the PDAC cells *versus* the A375 cells. Nonetheless, we evaluated a slightly higher CE slope in method M11 in an attempt to further improve peptide fragmentation as measured by PDI. HLA-I peptide identifications and allele assignments were maintained relative to M10 in melanoma samples while improving PDI across the different peptide charge states. The significant increase in precursor fragmentation for all charge states with M11 suggests that the use of the intermediate CE used in this method may be of more general use in the immunopeptidome analysis in larger patient cohorts that harbor diverse peptide-binding HLA alleles. In addition, a minor fraction of peptides (less than 2%) unique to M11 were longer (>9 amino acids) and present at higher charge state; therefore, this method could benefit samples containing HLA alleles that bind peptides containing basic amino acids. Accordingly, for the most comprehensive immunopeptidome depth and peptide representation, we recommend M11 for large patient cohort experiments.

A limitation of this study is that only Caucasian patient samples were included in the melanoma tumor cohort, which might bias the observed allele distribution or peptide features for which we optimized instrument data acquisition parameters. Based on this, we hypothesize that future studies with more comprehensive patient cohorts from diverse genetic backgrounds will provide further insights into improving immunopeptidome data acquisition parameters for diverse HLA allele populations. We believe that deep single-shot immunopeptidome datasets generated on the SCP will improve HLA binding and presentation prediction algorithms that are presently trained using predominantly ion trap and Orbitrap data (33, 50, 51, 52). Similarly, CCS prediction has been shown to increase confidence in low abundant peptide identifications of complex samples, but so far, it has been mainly trained on tryptic peptides (27). We therefore speculate that the generation of comprehensive HLA-I and HLA-II datasets to train CCS prediction algorithms on the unique features of HLA peptides will greatly benefit analysis of immunopeptidome data depth and help to improve the ability to directly identify low-level clinically relevant HLA antigens, such as neoantigens. Finally, stochastic sampling by the MS systems and, possibly, background introduced by the antibody enrichment continues to limit overlap between repeated single-shot injections of HLA-I-enriched samples to ca. 80%. Based on the reduced variability between replicates on the SCP relative to Exploris + FAIMS, we believe that the implementation of data-independent acquisition on the timsTOF instruments will improve sensitivity and specificity similar to recent advances on Orbitrap platforms (53, 54, 55). Moreover, we speculate that the improved dynamic range of the recently introduced high-throughput TIMS cartridge (timsTOF HT) in combination with rapid data-independent analysis, PASEF acquisition could greatly benefit data reproducibility and more efficiently sample low abundant peptide species. In summary, we believe that the SCP method optimization and systematic analyses presented here will enable deep immunopeptidome profiling of large patient cohorts, which in turn will facilitate improvement to HLA peptide presentation and CCS prediction algorithms.

## Data Availability

The original mass spectra, peptide spectrum match results, and the protein sequence databases used for searches have been deposited in the public proteomics repository MassIVE (http://massive.ucsd.edu) and are accessible at ftp://massive.ucsd.edu/MSV000091456/.

## Supplemental data

This article contains supplemental data.

## Conflict of interest

S. A. C. is a member of the scientific advisory boards of Kymera, PTM BioLabs, Seer, and PrognomIQ. A.S.V.J. is an employee of Bruker. All other authors declare no competing interests.
