# Supplementary material for: Sensitive, High-Throughput HLA-I and HLA-II Immunopeptidomics Using Parallel Accumulation-Serial Fragmentation Mass Spectrometry
Source: Mol Cell Proteomics. 2023 May 3;22(6):100563. doi: 10.1016/j.mcpro.2023.100563 (PMC10326702; doi:10.1016/j.mcpro.2023.100563)
Supplement: Supplemental Figures S1–S10 [file mmc1.pdf]

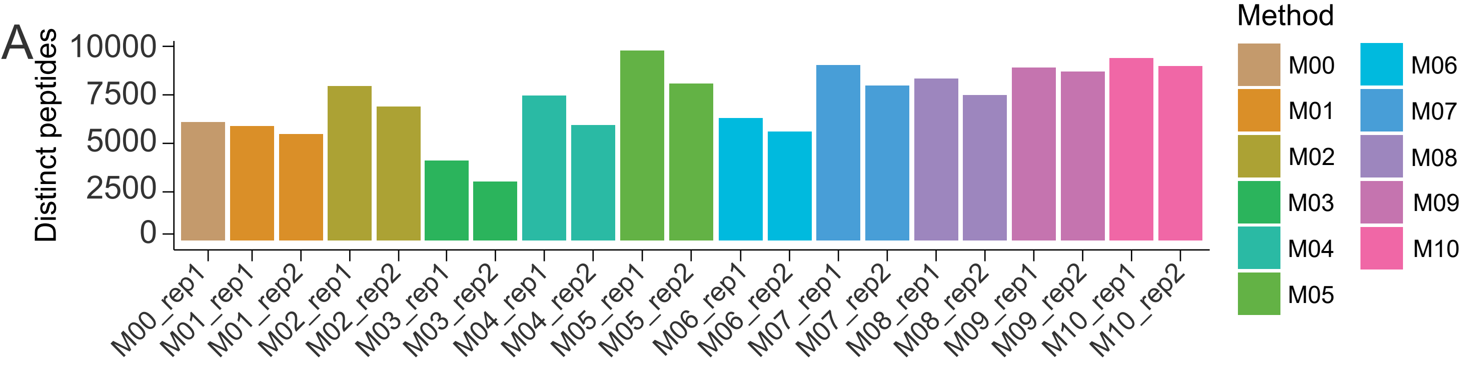


B

| directory | CE | HLA-I peptides | Score median | % SPI median | % BCS median | PDI median [+1] | PDI median [+2] | PDI median [+3] | Score median [+1] | Score median [+2] | Score median [+3] |
|---|---|---|---|---|---|---|---|---|---|---|---|
| M00 - rep1 | 20-59 | 6447 | 8.2 | 68.7 | 6.0 | 67.3 | 93.2 | 95.7 | 12.1 | 8.2 | 7.0 |
| M01 - rep1 | 20-59 | 6188 | 8.6 | 70.3 | 6.0 | 69.9 | 93.4 | 95.6 | 9.4 | 8.7 | 7.3 |
| M01 - rep2 | 20-59 | 5726 | 8.5 | 70.0 | 6.0 | 76.2 | 93.9 | 95.9 | 8.3 | 8.7 | 7.2 |
| M02 - rep1 | 20-59 | 8361 | 8.7 | 70.8 | 6.0 | 95.3 | 93.2 | 95.6 | 8.8 | 8.7 | 7.3 |
| M02 - rep2 | 20-59 | 7199 | 8.6 | 70.8 | 6.0 | 96.4 | 93.8 | 95.7 | 8.7 | 8.7 | 7.1 |
| M03 - rep1 | 30-65 | 4438 | 7.3 | 68.3 | 6.0 | 98.1 | 97.0 | 97.7 | 8.1 | 6.9 | 5.8 |
| M03 - rep2 | 30-65 | 3343 | 7.1 | 67.9 | 6.0 | 98.5 | 97.1 | 97.7 | 7.6 | 6.8 | 5.5 |
| M04 - rep1 | Stepped | 7791 | 8.4 | 70.0 | 6.0 | 98.0 | 93.2 | 95.7 | 8.0 | 8.6 | 7.2 |
| M04 - rep2 | Stepped | 6244 | 8.3 | 70.1 | 6.0 | 98.4 | 93.9 | 95.7 | 8.1 | 8.5 | 7.1 |
| M05 - rep1 | 10-55 | 10130 | 9.7 | 72.7 | 6.0 | 91.1 | 69.7 | 65.1 | 9.2 | 10.3 | 8.0 |
| M05 - rep2 | 10-55 | 8409 | 9.9 | 73.4 | 6.0 | 92.6 | 70.3 | 66.0 | 9.3 | 10.3 | 8.0 |
| M06 - rep1 | 20-59 | 6538 | 8.2 | 69.0 | 6.0 | 96.0 | 92.9 | 95.4 | 8.5 | 8.1 | 6.8 |
| M06 - rep2 | 20-59 | 6024 | 8.2 | 69.1 | 6.0 | 95.9 | 93.5 | 95.5 | 8.5 | 8.1 | 6.7 |
| M07 - rep1 | 20-59 | 9468 | 8.8 | 71.2 | 6.0 | 95.5 | 92.9 | 95.2 | 8.9 | 8.7 | 7.5 |
| M07 - rep2 | 20-59 | 8292 | 8.8 | 71.2 | 6.0 | 95.7 | 93.4 | 95.7 | 9.1 | 8.7 | 7.3 |
| M08 - rep1 | 20-59 | 8519 | 9.9 | 75.7 | 6.0 | 96.0 | 93.8 | 95.6 | 9.8 | 10.0 | 8.1 |
| M08 - rep2 | 20-59 | 7576 | 9.9 | 76.0 | 6.0 | 96.1 | 94.1 | 95.9 | 9.7 | 10.0 | 8.2 |
| M09 - rep1 | 10-55 | 9162 | 10.1 | 73.9 | 6.0 | 91.9 | 71.0 | 68.0 | 9.4 | 10.5 | 8.0 |
| M09 - rep2 | 10-55 | 9063 | 10.0 | 73.9 | 6.0 | 91.5 | 70.0 | 65.1 | 9.4 | 10.5 | 8.0 |
| M10 - rep1 | 10-55 | 9493 | 11.3 | 78.4 | 6.0 | 92.1 | 70.7 | 64.2 | 10.5 | 12.0 | 8.8 |
| M10 - rep2 | 10-55 | 9001 | 11.4 | 78.5 | 6.0 | 91.5 | 69.2 | 62.6 | 10.3 | 12.1 | 9.0 |

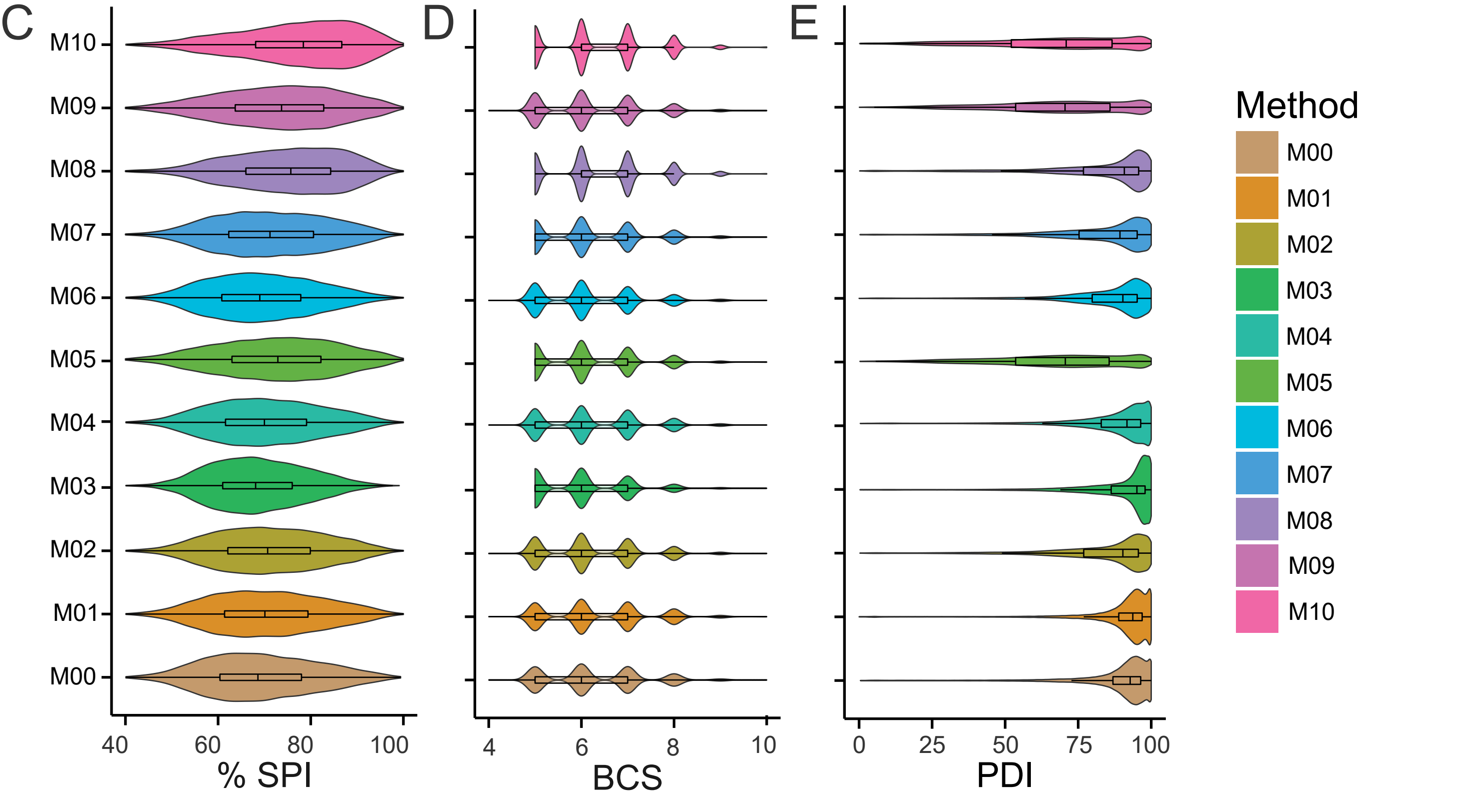

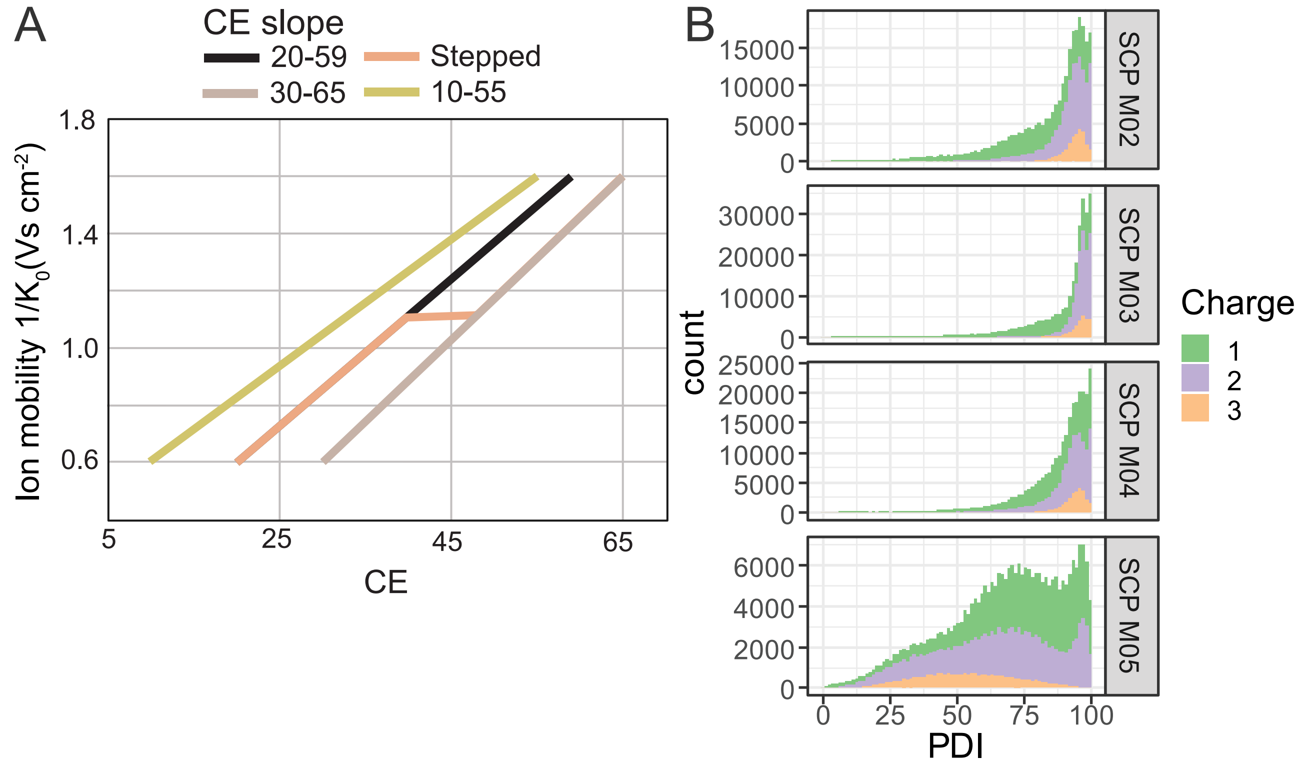
A
CE slope
20-59
Stepped
30-65
10-55
Ion mobility 1/$K_0$(Vs $cm^{-2}$)
1.8
1.4
1.0
0.6
5
25
45
65
CE
B
count
SCP M02
SCP M03
SCP M04
SCP M05
15000
10000
5000
0
30000
20000
10000
0
25000
20000
15000
10000
5000
0
6000
4000
2000
0
0
25
50
75
100
PDI
Charge
1
2
3

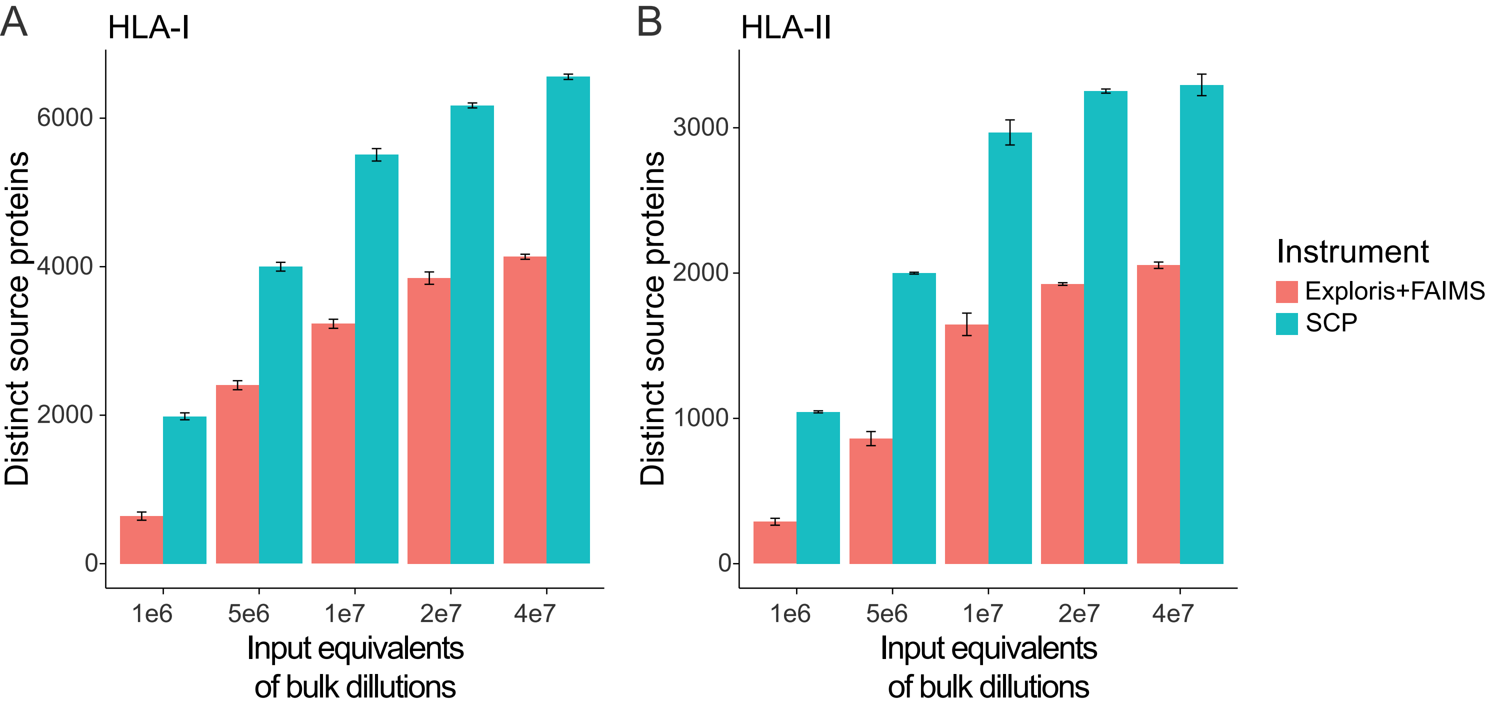
A
HLA-I
Distinct source proteins
6000
4000
2000
0
1e6
5e6
1e7
2e7
4e7
Input equivalents
of bulk dillutions
B
HLA-II
Distinct source proteins
3000
2000
1000
0
1e6
5e6
1e7
2e7
4e7
Input equivalents
of bulk dillutions
Instrument
Exploris+FAIMS
SCP

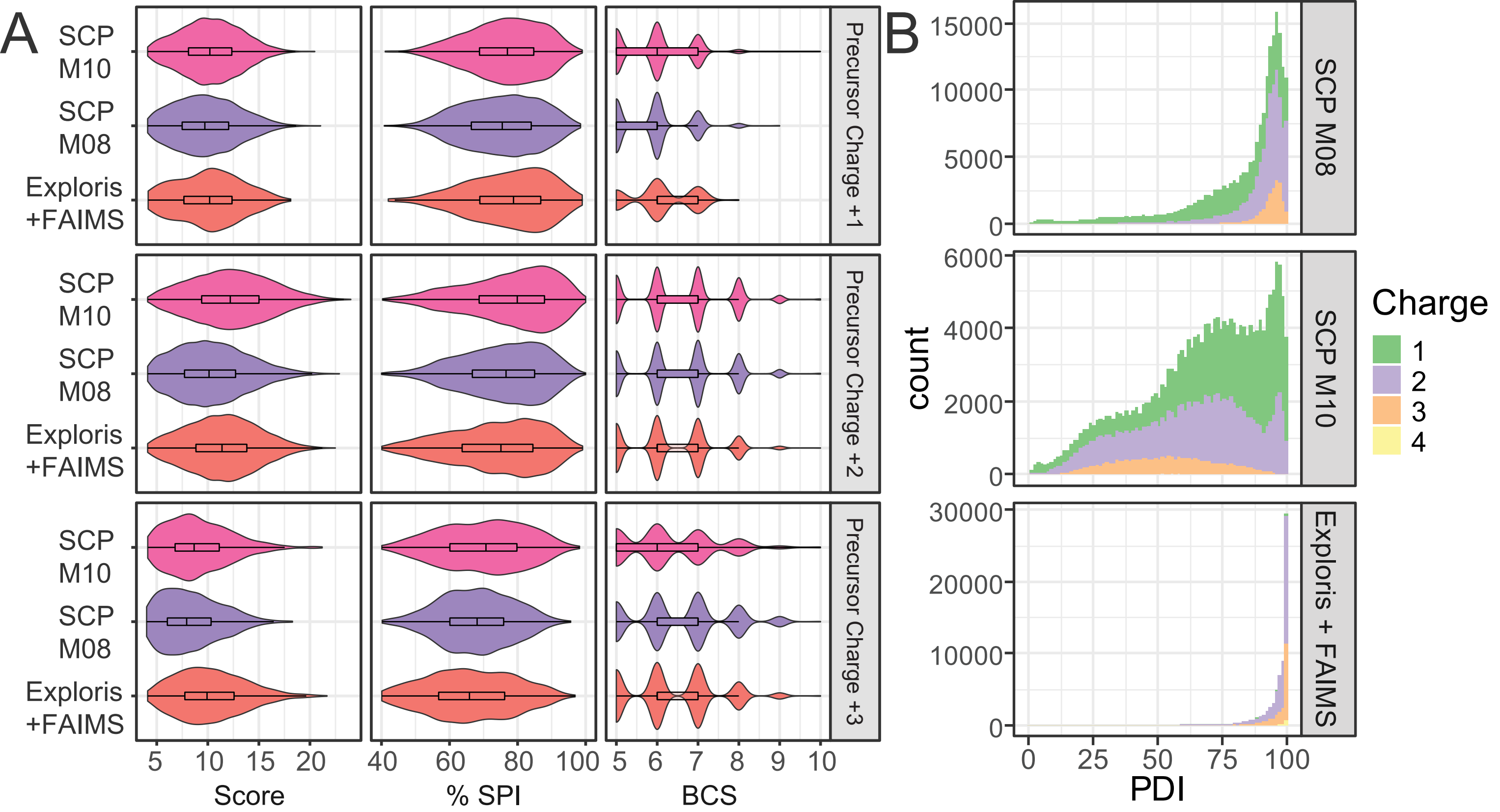


C

| Sample | Score median | % SPI median | % BCS median | PDI median [+1] | PDI median [+2] | PDI median [+3] | PDI median [+4] |
|---|---|---|---|---|---|---|---|
| SCP M10 | 11.3 | 78.4 | 6.0 | 92.1 | 70.7 | 64.2 | N/A |
| SCP M08 | 9.9 | 75.7 | 6.0 | 96.0 | 93.8 | 95.6 | N/A |
| Exploris+FAIMS | 10.63 | 75.9 | 6.0 | N/A | 98 | 98.1 | 97.6 |

A

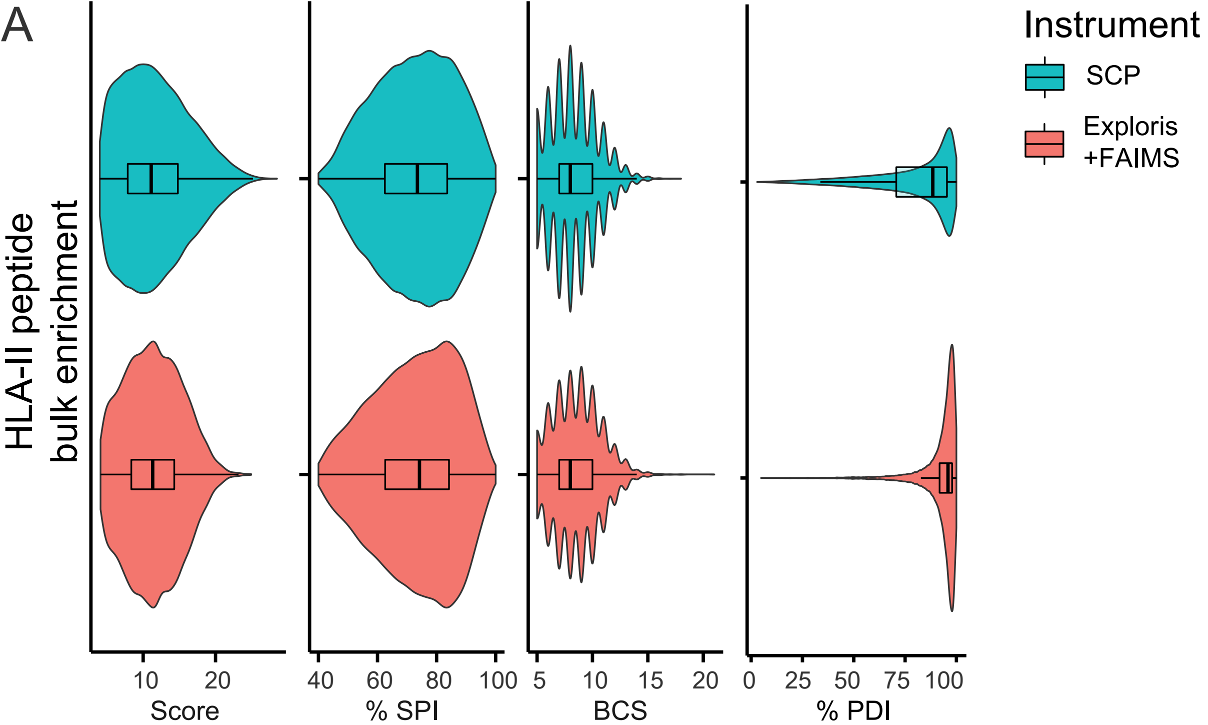


B

| Sample | Score median | % SPI median | % BCS median | PDI median [+2] | PDI median [+3] | PDI median [+4] | PDI median [+5] |
|---|---|---|---|---|---|---|---|
| SCP | 10.61 | 71.1 | 8 | 75.2 | 91.8 | 95.7 | 96.75 |
| Exploris+FAIMS | 10.85 | 74.9 | 8.5 | 98.8 | 98.9 | 98.9 | 98.6 |

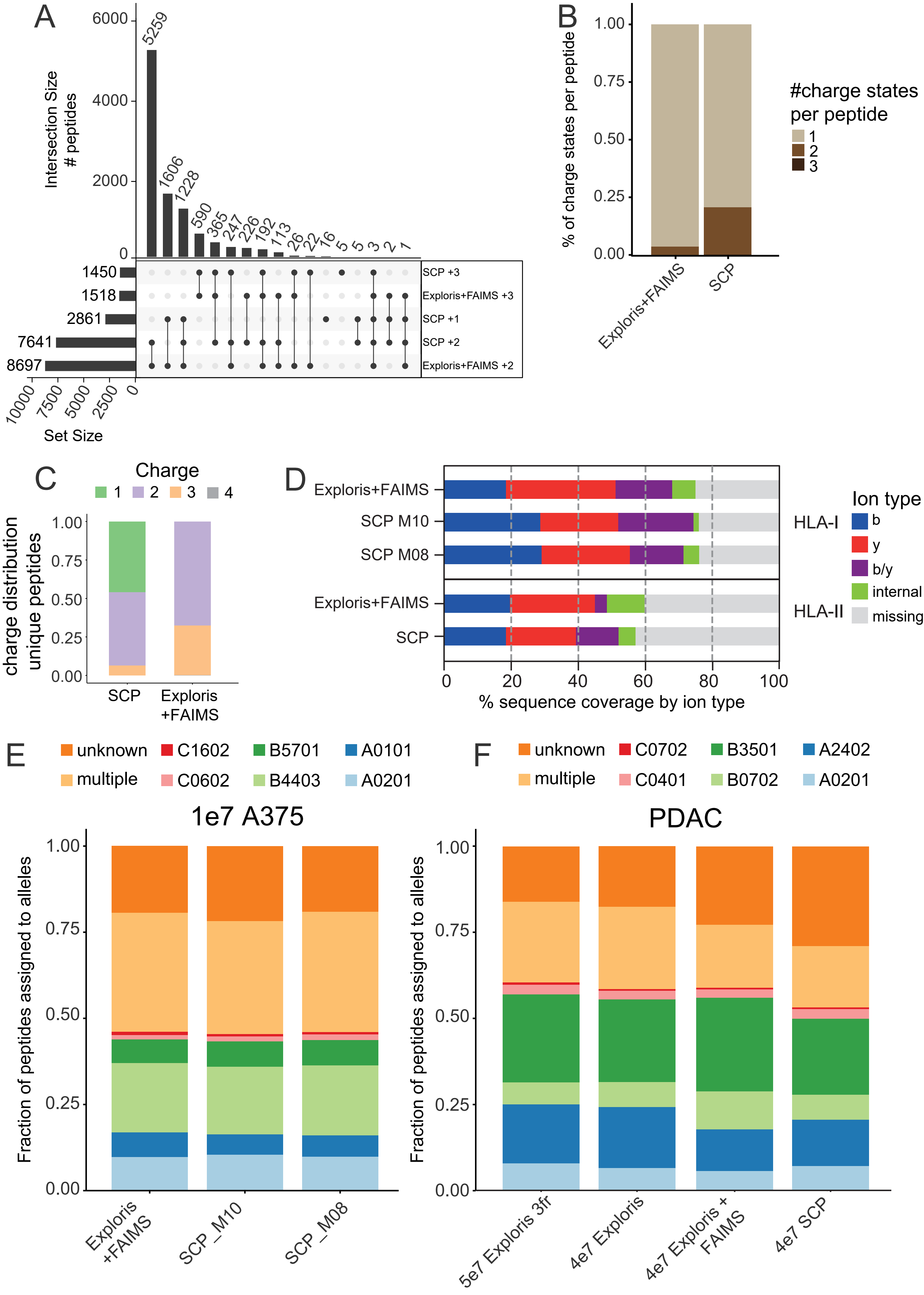
A
Intersection Size
# peptides
6000
4000
2000
0
5259
1606
1228
590
365
247
226
192
113
26
22
16
5
5
3
2
1
1450
1518
2861
7641
8697
SCP +3
Exploris+FAIMS +3
SCP +1
SCP +2
Exploris+FAIMS +2
10000
7500
5000
2500
0
Set Size
B
% of charge states per peptide
1.00
0.75
0.50
0.25
0.00
Exploris+FAIMS
SCP
#charge states
per peptide
1
2
3
C
Charge
1
2
3
4
charge distribution
unique peptides
1.00
0.75
0.50
0.25
0.00
SCP
Exploris
+FAIMS
D
Exploris+FAIMS
SCP M10
SCP M08
Exploris+FAIMS
SCP
HLA-I
HLA-II
Ion type
b
y
b/y
internal
missing
0
20
40
60
80
100
% sequence coverage by ion type
E
unknown
C1602
B5701
A0101
multiple
C0602
B4403
A0201
1e7 A375
Fraction of peptides assigned to alleles
1.00
0.75
0.50
0.25
0.00
Exploris
+FAIMS
SCP_M10
SCP_M08
F
unknown
C0702
B3501
A2402
multiple
C0401
B0702
A0201
PDAC
Fraction of peptides assigned to alleles
1.00
0.75
0.50
0.25
0.00
5e7 Exploris 3fr
4e7 Exploris
4e7 Exploris +
FAIMS
4e7 SCP

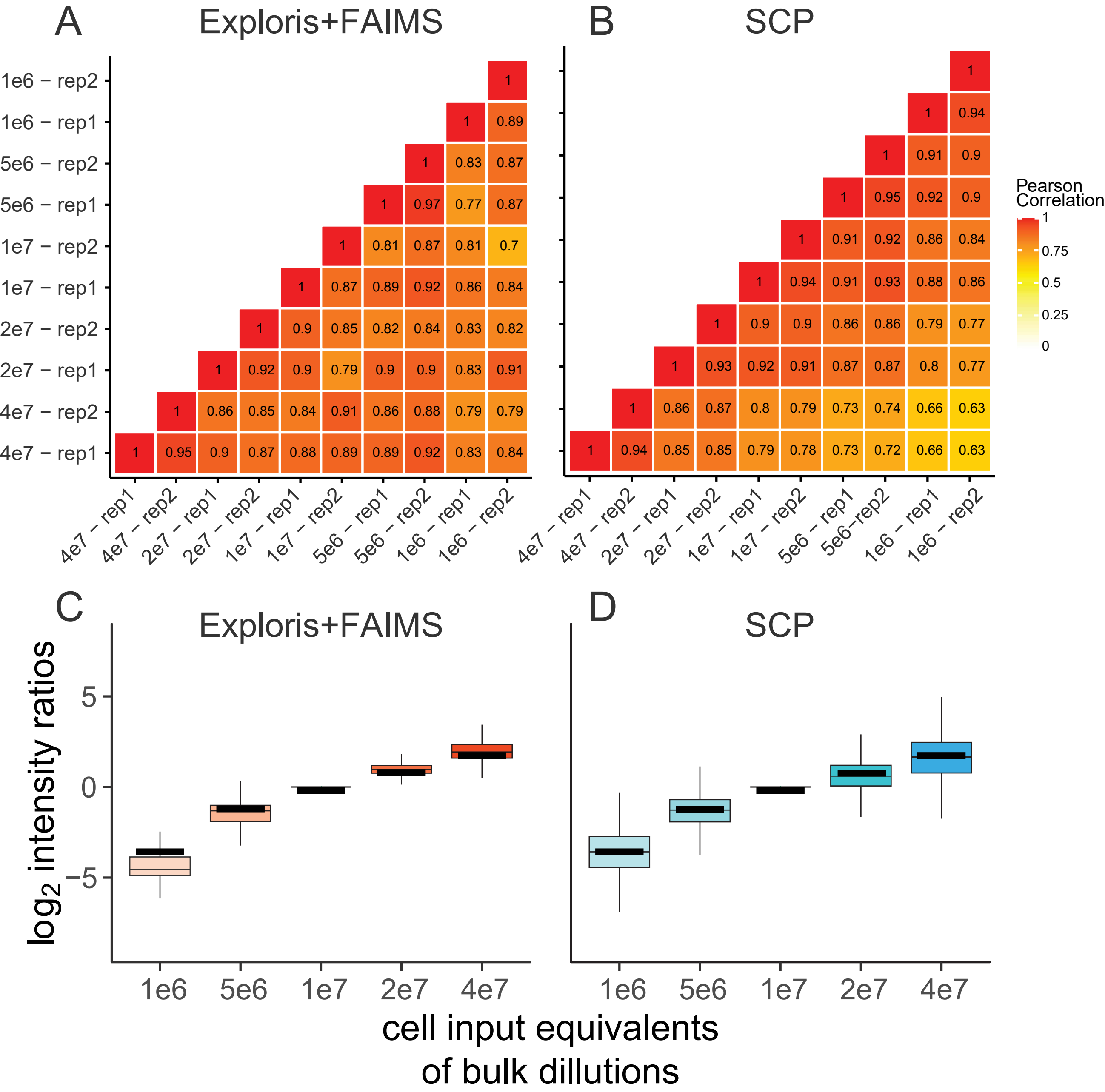
A
Exploris+FAIMS
B
SCP
1e6 − rep2
1e6 − rep1
5e6 − rep2
5e6 − rep1
1e7 − rep2
1e7 − rep1
2e7 − rep2
2e7 − rep1
4e7 − rep2
4e7 − rep1
Pearson Correlation
1
0.75
0.5
0.25
0
C
Exploris+FAIMS
D
SCP
log2 intensity ratios
5
0
−5
1e6
5e6
1e7
2e7
4e7
cell input equivalents
of bulk dillutions

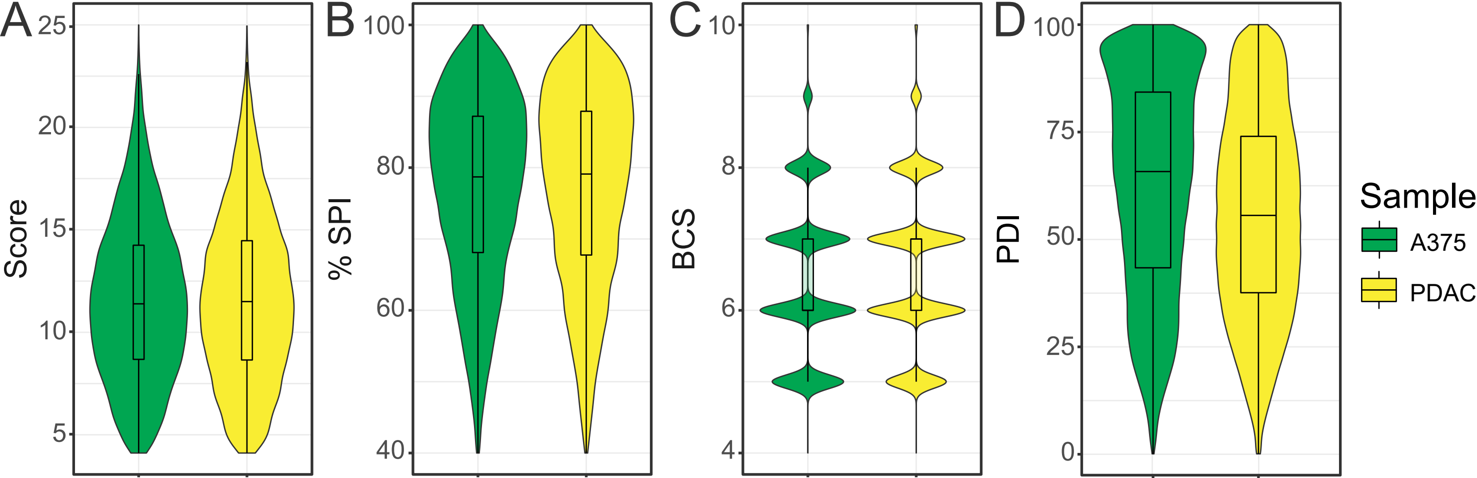

A
B
C
D
Score
% SPI
BCS
PDI
25
20
15
10
5
100
80
60
40
10
8
6
4
100
75
50
25
0
Sample
A375
PDAC

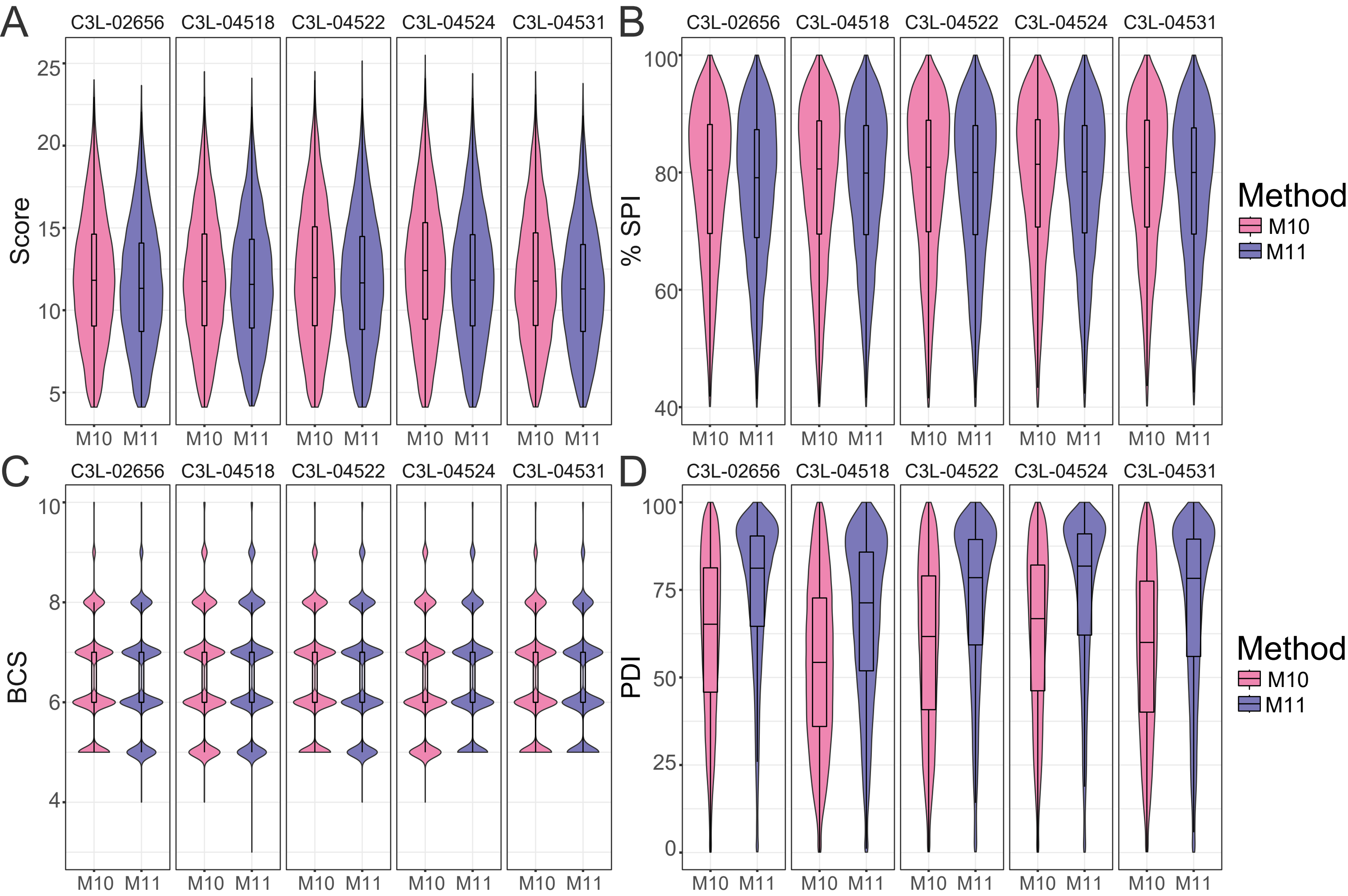

A
B
C
D
C3L-02656
C3L-04518
C3L-04522
C3L-04524
C3L-04531
Score
% SPI
BCS
PDI
M10
M11
Method

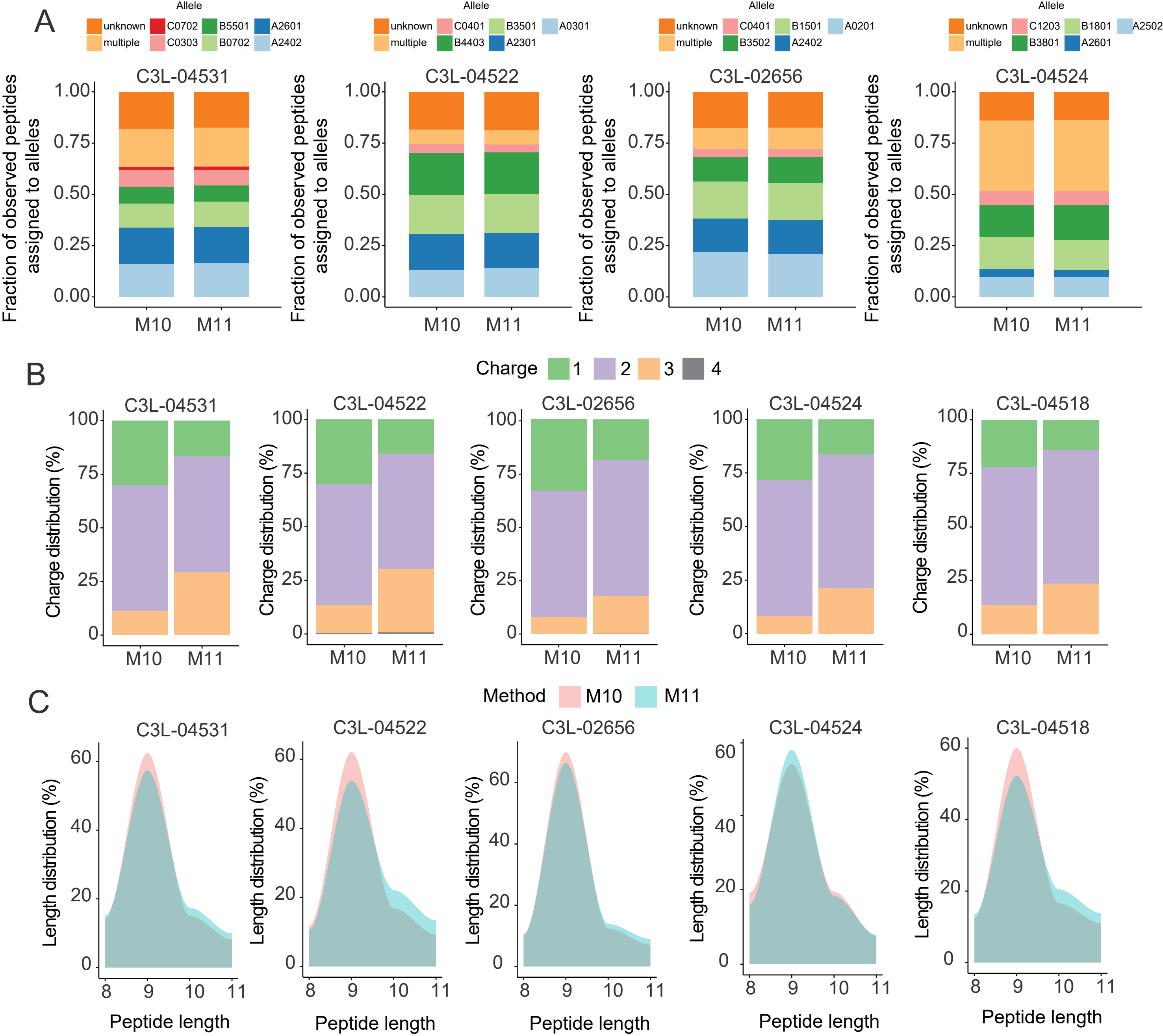

A
Allele
unknown
multiple
C0702
C0303
B5501
B0702
A2601
A2402
C0401
B4403
B3501
A2301
A0301
C0401
B3502
B1501
A2402
A0201
C1203
B3801
B1801
A2601
A2502
C3L-04531
C3L-04522
C3L-02656
C3L-04524
Fraction of observed peptides assigned to alleles
M10
M11
B
Charge
1
2
3
4
C3L-04518
Charge distribution (%)
C
Method
M10
M11
Length distribution (%)
Peptide length
