# Supplementary material for: Sensitive, High-Throughput HLA-I and HLA-II Immunopeptidomics Using Parallel Accumulation-Serial Fragmentation Mass Spectrometry
Source: Mol Cell Proteomics. 2023 May 3;22(6):100563. doi: 10.1016/j.mcpro.2023.100563 (PMC10326702; doi:10.1016/j.mcpro.2023.100563)
Supplement: Supplemental Figures S1–S10 and Tables S1–S10 [file mmc2.docx]

**Supplemental Figure legends**

**Supplemental Figure S1: Quality metrics of HLA-I peptides from A375 cells identified with different acquisition methods on the timsTOF SCP.**

A, Unique A375 HLA-I peptides identified from per injection replicate for M00-M10 on the timsTOF SCP.

B, Quality metrics for HLA-I peptides from A375 cells as calculated by SpectrumMill including median score, % SPI, BCS, PDI and scores by charge state using M00-M10.

C, Distribution of % SPI of A375 HLA-I peptides identified with M00-M10.

D, BCS distribution of HLA-I peptides identified with M00-M10.

E, PDI across all identified HLA-I peptides using M00-M10.

% SPI is percent scored peak intensity, BCS is backbone cleavage score, PDI is percent precursor dissociation intensity.

**Supplemental Figure 2: CE dependency to IM impacts HLA-I peptide fragmentation on the SCP.**

A, Representative CE slopes 20-59 (M00-M02, M06-M08), 30-65 (M03), 10-55 (M05, M09-M10) from 1/K0 = 0.6 Vs cm^-2^ to 1/K0 = 1.6 Vs cm^-2^ or stepped (20-40 from 1/K0 = 0.6-1.1 Vs cm^-2^ and 48-65 from 1/K0 = 1.1-1.6 Vs cm^-2^).

B, PDI on the timsTOF SCP with M02-M05 by charge state.

CE is collisional energy, PDI is percent precursor dissociation intensity.

**Supplemental Figure 3: Single-shot acquisition of HLA-I and HLA-II peptides on the timsTOF SCP increases source protein coverage >1.5-fold compared to Exploris + FAIMS.**

A, Unique source proteins represented in HLA-I immunopeptidomes by single injections on Exploris + FAIMS (red) and timsTOF SCP (blue). Mean and standard deviation is shown.

B, Unique source proteins represented in HLA-II immunopeptidomes by single injections on Exploris + FAIMS (red) and timsTOF SCP (blue). Mean and standard deviation is shown.

**Supplemental Figure 4: Quality metrics of HLA-I peptides identified on the timsTOF SCP upon CE alterations and the Exploris + FAIMS.**

A, Distributions of score, % SPI and BCS of HLA-I peptides on the timsTOF SCP with M10 (pink), M08 (purple) or the Exploris + FAIMS (orange) by charge state.

B, PDI on the timsTOF SCP with M08, M10 or the Exploris + FAIMS by charge state.

C, Quality metrics for HLA-I peptides as calculated by SpectrumMill including median score, % SPI, BCS, median PDI by charge state for the timsTOF SCP (M10 and M08) or the Exploris + FAIMS.

CE is collisional energy, % SPI is percent scored peak intensity, BCS is backbone cleavage score, PDI is percent precursor dissociation intensity. ‘N/A’ indicates that the defined acquisition method does not include these charge states.

**Supplemental Figure 5: Quality metrics of HLA-II peptides identified on the timsTOF SCP and the Exploris + FAIMS are highly comparable.**

A, Distributions of score, % SPI, BCS and PDI of HLA-II peptides identified on the timsTOF SCP and Exploris + FAIMS.

B, Quality metrics for HLA-II peptides as calculated by SpectrumMill including median score, % SPI, BCS, median PDI by charge state for the SCP or the Exploris + FAIMS.

% SPI is percent scored peak intensity, BCS is backbone cleavage score, PDI is percent precursor dissociation intensity.

**Supplemental Figure 6: Charge state distribution, sequence coverage by ion type and allele assignments of HLA-I and HLA-II peptides identified on the timsTOF SCP or Exploris ± FAIMS.**

A, Overlap of HLA-I peptides identified on both instruments in charge states 1, 2 or 3 on the timsTOF SCP or in charge states 2 and 3 on the Exploris + FAIMS.

B, Percentage of commonly identified HLA-I peptides on both the timsTOF SCP or Exploris + FAIMS in a single (beige), two (brown) or three (dark brown) charge states per peptide.

C, Percentage of uniquely identified HLA-I peptides on both the SCP or the Exploris + FAIMS per charge state.

D, Percent sequence coverage by ion type for HLA-I and HLA-II peptides from the timsTOF SCP or the Exploris + FAIMS. b-ions in blue, y-ions in red, b/y-ion pairs in purple, internal-ions in green and missing/unassigned-ions in gray.

E, Allele assignment of 8-11 aa long HLA-I peptides identified on Exploris + FAIMS or SCP (M10 and M08), filtered for HLAthena rank <0.5 in 1e7 A375 cells.

F, Allele assignment of 8-11 aa long HLA-I peptides identified from the PDAC cell line across 3 fractions (3fr) or single-shot injections on the Exploris, Exploris + FAIMS and the timsTOF SCP. Data is filtered for HLAthena rank <0.5.

PDAC is patient derived adenocarcinoma cells.

**Supplemental Figure 7: HLA-I analysis on the timsTOF SCP shows comparable reproducibility to Exploris + FAIMS.**

A, Intensity correlation of HLA-I peptides between technical replicates on the Exploris + FAIMS.

B, Intensity correlation between technical replicates of HLA-I peptides identified on the SCP.

C, Log_2_ intensity ratios at indicated input equivalents relative to 1e7 cell equivalents of bulk digests on the Exploris + FAIMS. Horizontal black line indicates theoretical ratio.

D, Log_2_ intensity ratios at indicated input equivalents relative to 1e7 cell equivalents of bulk digests on the SCP. Horizontal black line indicates theoretical ratio.

**Supplemental Figure 8: PDI of HLA-I peptides identified on the timsTOF SCP differs between A375 and PDAC cell lines.**

A, Score distribution of HLA-I peptides from A375 cells (green) or PDAC line (yellow) on the timsTOF SCP. B, %SPI of HLA-I peptides from A375 cells (green) or PDAC line (yellow) on the timsTOF SCP.

C, BCS of HLA-I peptides from A375 cells (green) or PDAC line (yellow) on the timsTOF SCP.

D, PDI of HLA-I peptides from A375 cells (green) or PDAC line (yellow) on the timsTOF SCP.

PDAC is pancreatic ductal adenocarcinoma tumor cell.

**Supplemental Figure 9: CE alterations on the SCP improves PDI without impacting scores of HLA-I peptides from primary melanoma tumors.**

A, Score distribution of HLA-I peptides of HLA-I peptides from primary melanoma tumors upon CE alterations (M10 or M11) acquired on the timsTOF SCP.

B, % SPI of HLA-I peptides of HLA-I peptides from primary melanoma tumors upon CE alterations (M10 or M11) acquired on the timsTOF SCP.

C, BCS of HLA-I peptides of HLA-I peptides from primary melanoma tumors upon CE alterations (M10 or M11) acquired on the timsTOF SCP.

D, PDI of HLA-I peptides of HLA-I peptides from primary melanoma tumors upon CE alterations (M10 or M11) acquired on the timsTOF SCP.

**Supplemental Figure 10: CE alterations on the timsTOF SCP impact charge state distribution of identified HLA-I peptides from primary melanoma tumors.**

A, Allele assignment of HLA-I 8-11 mers identified on the timsTOF SCP with either M10 or M11, filtered for HLAthena rank <0.5 in primary melanoma tumors.

B, Charge state distribution of HLA-I peptides from M10 or M11 on the timsTOF SCP.

C, Peptide length distribution across identified HLA-I peptides on the timsTOF SCP with M10 or M11 in primary melanoma tumors.

CE is collisional energy.

**Supplementary Table 1:** Spectrum Mill peptide export of HLA-I peptides identified with methods M00-M10 using different acquisition parameters on the timsTOF SCP. Table includes score, % SPI, BCS and other metrics from bulk enriched HLA-I peptides from A375 cells diluted to 1e7 cell input equivalents and is filtered for common contaminants, background binders, tryptic peptides and ± 10 ppm mass error.

**Supplementary Table 2:** Spectrum Mill peptide export including score, % SPI, BCS and other metrics from bulk HLA-I enriched A375 cells diluted to 1e6 to 4e7 cell input equivalents acquired on the Exploris + FAIMS. Table is filtered for length (8-11aa), common contaminants, background binders, tryptic peptides and ± 10 ppm mass error. MS1 quantification from IonQuant within the Fragpipe environment is appended.

**Supplementary Table 3:** Spectrum Mill peptide export including score, % SPI, BCS and other metrics from bulk HLA-I enriched A375 cells diluted to 1e6 to 4e7 cell input equivalents acquired on the timsTOF SCP. Table is filtered for length (8-11aa), common contaminants, background binders, tryptic peptides and ± 10 ppm mass error. MS1 quantification from IonQuant within the Fragpipe environment is appended.

**Supplementary Table 4:** Spectrum Mill peptide export including score, % SPI, BCS and other metrics from low-input (i.e. 1e6 to 4e7) HLA-I enriched A375 cells acquired on the timsTOF SCP. Table is filtered for length (8-11aa), common contaminants, background binders, tryptic peptides and ± 10 ppm mass error.

**Supplementary Table 5:** Spectrum Mill peptide export including score, BCS, %SPI and other MS identification metrics of HLA-II peptides bulk enriched from A375 cells and loaded at different dilutions on Exploris+FAIMS. Peptides were filtered for length (8-60aa), common contaminants, background binders, tryptic peptide contamination and ± 10ppm mass error.

**Supplementary Table 6:** Spectrum Mill peptide export including score, BCS, %SPI and other MS identification metrics of HLA-II peptides bulk enriched from A375 cells and loaded at different dilutions on the timsTOF SCP. Peptides were filtered for length (8-60aa), common contaminants, background binders, tryptic peptide contamination and ± 10ppm mass error.

**Supplementary Table 7:** Spectrum Mill peptide export including score, BCS, %SPI and other MS identification metrics of HLA-I peptides enriched patient derived PDAC cell line and either brp fractionated into 3 fractions or loaded in single shot on the Exploris, Exploris+FAIMS or timsTOF SCP as indicated in column “directory”. Peptides were filtered for length (8-11aa), common contaminants, background binders, tryptic peptide contamination and ± 10ppm mass error. “Species” column indicates whether peptides were identified from annotated human proteins, nuORFs or both. A separate tab “CTA_PDAC” contains a smaller subset of these peptides which are derived from CTA source proteins.

**Supplementary Table 8:** Spectrum Mill peptide export including score, BCS, %SPI and other MS identification metrics of a subset of HLA-I peptides from A375 and PDAC cells that are derived from nuORFs and further subjected to stringent subset FDR filtering as detailed in “Subset-specific FDR filtering for nuORFs” in the Experimental Procedure section. The “directory” column indicates the corresponding cell line, input amount, experiment and acquisition scheme.

**Supplementary Table 9:** Spectrum Mill peptide export including score, BCS, %SPI and other MS identification metrics of HLA-I peptides enriched from primary melanoma tumors and loaded in single shot on the timsTOF SCP, with the patient ID indicated in column “directory”. Peptides were filtered for length (8-11aa), common contaminants, background binders, tryptic peptide contamination and ± 10ppm mass error. “Species” column indicates whether peptides were identified from annotated human proteins, nuORFs or both. A separate tab “CTA_Melanoma” contains a smaller subset of these peptides which are derived from CTA source proteins.

**Supplementary Table 10:** Spectrum Mill peptide export including score, BCS, %SPI and other MS identification metrics of a subset of HLA-I peptides from primary melanoma tumors reported in Table S9 that are derived from nuORFs and further subjected to stringent subset FDR filtering as detailed in “Subset-specific FDR filtering for nuORFs” in the Experimental Procedure section. The “directory” column indicates the patient ID.
